# Binding of the transcription activator-like effector augments transcriptional regulation by another transcription factor

**DOI:** 10.1093/nar/gkac454

**Published:** 2022-06-07

**Authors:** Katja Leben, Žiga Strmšek, Tina Lebar, Anže Verbič, Matej Dragovan, Neža Omersa, Gregor Anderluh, Roman Jerala

**Affiliations:** Department of Synthetic Biology and Immunology, National Institute of Chemistry, Hajdrihova 19, SI-1000 Ljubljana, Slovenia; Interdisciplinary Doctoral Programme in Biomedicine, University of Ljubljana, Kongresni trg 12, SI-1000 Ljubljana, Slovenia; Department of Synthetic Biology and Immunology, National Institute of Chemistry, Hajdrihova 19, SI-1000 Ljubljana, Slovenia; Department of Synthetic Biology and Immunology, National Institute of Chemistry, Hajdrihova 19, SI-1000 Ljubljana, Slovenia; Department of Synthetic Biology and Immunology, National Institute of Chemistry, Hajdrihova 19, SI-1000 Ljubljana, Slovenia; Interdisciplinary Doctoral Programme in Biomedicine, University of Ljubljana, Kongresni trg 12, SI-1000 Ljubljana, Slovenia; Department of Synthetic Biology and Immunology, National Institute of Chemistry, Hajdrihova 19, SI-1000 Ljubljana, Slovenia; Department of Molecular Biology and Nanobiotechnology, National Institute of Chemistry, Hajdrihova 19, SI-1000 Ljubljana, Slovenia; Department of Molecular Biology and Nanobiotechnology, National Institute of Chemistry, Hajdrihova 19, SI-1000 Ljubljana, Slovenia; Department of Synthetic Biology and Immunology, National Institute of Chemistry, Hajdrihova 19, SI-1000 Ljubljana, Slovenia

## Abstract

DNA transcription is regulated by a range of diverse mechanisms and primarily by transcription factors that recruit the RNA polymerase complex to the promoter region on the DNA. Protein binding to DNA at nearby or distant sites can synergistically affect this process in a variety of ways, but mainly through direct interactions between DNA-binding proteins. Here we show that a Transcription Activator-Like Effector (TALE), which lacks an activation domain, can enhance transcription in mammalian cells when it binds in the vicinity of and without direct interaction with several different dimeric or monomeric transcription factors. This effect was observed for several TALEs regardless of the recognition sequences and their DNA-bound orientation. TALEs can exert an effect over the distance of tens of nucleotides and it also potentiated KRAB-mediated repression. The augmentation of transcriptional regulation of another transcription factor is characteristic of TALEs, as it was not observed for dCas9/gRNA, zinc finger, or Gal4 DNA-binding domains. We propose that this mechanism involves an allosteric effect exerted on DNA structure or dynamics. This mechanism could be used to modulate transcription but may also play a role in the natural context of TALEs.

## INTRODUCTION

Cell type and activity depend on combinations of genes expressed or suppressed via various mechanisms of transcriptional regulation. A variety of signals affect gene expression in cells, and this process is regulated by diverse transcription factors acting in concert (1,2). Interactions between protein regulators are driven by several mechanisms, including direct and cooperative interactions (2,3). While direct interactions between monomeric or oligomeric transcription factors and DNA have been extensively studied (4), recent evidence suggests that DNA-mediated cooperativity is an important factor in transcriptional regulation. DNA can facilitate protein interactions between proteins that interact weakly in the absence of DNA (5–7), or even mediate interactions between proteins that are not in direct contact. In the latter case, binding of one protein can alleviate binding constraints on the other through allosteric changes transmitted via DNA (8–11). A protein can facilitate binding to the second binding site by distorting the structure of DNA (indirect readout mechanism), inducing changes in water or ion distribution (solvent release mechanism), or quenching the vibrational modes of DNA (entropy-mediated mechanism) (9). Whereas direct interactions between proteins are highly specific and the two binding partners must be close to each other to interact, allosteric cooperativity is less specific in terms of binding partners and the range varies from short for DNA-facilitated and DNA-mediated interactions to tens of base pairs for entropy-mediated changes. Research on cooperative interactions is particularly important in mammalian cells, where transcription occurs in dense clusters that span large distances (8).

Transcriptional regulation is of particular interest to synthetic biology as means for controlling cellular processes (12). DNA-binding proteins that influence transcription are a key tool in the synthetic biologist's toolbox. The more diverse these tools are, the more precise circuits can be constructed, resembling versatile natural regulation of transcription (13). The discovery of proteins with designable DNA-binding properties, such as transcription activator-like effectors (TALEs), has propelled synthetic biology forward in its search for modular components to design synthetic regulatory pathways composed of modular building blocks that are orthogonal to other cellular functions and can target in principle any DNA sequence of interest (13).

TALE proteins originate from the genus *Xanthomonas*, bacteria that secrete TALEs into cells of their plant host, impairing their function, eliciting disease, and hindering plant defense responses. Due to their specificity and designability, they are important for synthetic gene regulation. Structurally, a TALE protein can be divided into three domains: N-terminal domain (NTD), central repeat region (CRR) and C-terminal domain (CTD). TALE binds to DNA in a multipartite manner: The NTD binds DNA in a less specific manner and plays a role in DNA recognition (14), and the CRR provides the designable DNA-binding site of a protein and is key to DNA-binding specificity. This combination enables a unique rotationally decoupled search mechanism (15). The CRR consists of canonical repeats of 33–35 amino acid residues with conserved sequence, except for amino acid residues at positions 12 and 13, termed repeat variable diresidue (RVD). Each canonical repeat forms a hairpin positioning amino acid residues at positions 12 and 13 in the proximity of the sense strand nucleotide, thereby determining base pair specificity. Since each canonical repeat recognizes a single base in a DNA sequence, the sequence of all RVDs determines the target site of a TALE protein (13,16). Collectively, all canonical repeats form a right-handed superhelix that binds strongly into a major groove (17,18).

Since the discovery of their potential for synthetic biology, TALE proteins have been used for various applications. To facilitate their use, the N- and C-terminal ends of the effector domains have been pruned to contain only those parts of the N- and C-terminal domains that are essential for proper DNA-binding and structure (13). The number of CRR repeats was optimized as well (19,20). Some of the most distinctive applications include genetic fusion with the Fok nuclease, which enables targeted genome editing (20), while effector domains such as the VP16 or VPR activation domains (1,21) or the KRAB silencing domain (21) have proven immensely useful in designing genetic circuits. Interestingly, TALE proteins are able to efficiently displace 3’-bound proteins from DNA in a polarized manner. This has been used to generate competitive repressors (22), to displace diverse 3’-bound DNA-binding proteins adjacent to TALE binding site (23), and to engineer genetic logic gates. The polarized displacement is based on steric hindrance and the multipartite NTD of TALE for DNA-binding, and this effect extended over a few base pairs from the binding site of the targeted DNA-binding protein.

Here, we report a novel feature of TALE proteins, which are able to enhance transcriptional regulation elicited by another transcriptional regulator, either as an activator or a repressor. The effect persists over tens of nucleotides, regardless of the orientation of the bound TALE. The enhancement effect was demonstrated for several types of transcription factors, whereas this enhancement is not exhibited by several other DNA-binding domains tested. We discuss the possible mode of action of this effect, which may contribute to our understanding of synergistic effects on DNA transcription.

## MATERIALS AND METHODS

### Plasmid construction

All plasmids were cloned using the restriction-ligation approach or Gibson assembly (24). All nucleotide and amino acid sequences are available in Supplementary materials. The distance of transcription factor target site to promoter is the same in all constructs used in the same experiment. Source of DNA sequences used in cloning is indicated in Table S1.

### Transcriptional activation reporter constructs

Reporter plasmids were constructed from pGL4.16[luc2CP/Hygro] vector (Promega) with luc2CP Firefly luciferase. Firefly luciferase was placed under minimal promoter and target sites for TALE and transcriptional activator were placed upstream. These sequences were cloned into pGL4.16 between ‘synthetic poly (A) signal/ transcriptional pause site’ and ‘luc2CP’ feature of pGL4.16 vector. DNA sequences are available in Table S2. A pGL4.16 with a gene encoding BFP instead of luc2CP was constructed. Amino acid sequence of BFP is available in Table S7.

### Transcriptional repression reporter constructs

Reporter plasmids were constructed from pcDNA3. Luc2CP from pGL4.16 was cloned between HindII and XbaI restriction sites, placing luc2CP Firefly luciferase under constitutive CMV promoter. Target sites for TALE and transcriptional activator were cloned upstream. DNA sequences of target sites cloned between AmpR promoter feature and CMV promoter are available in Table S3.

### DNA-binding protein constructs

Genes encoding DNA-binding proteins and their fusions to effector domains are under strong constitutive CMV promoter in pcDNA3 (Invitrogen). Amino acid sequences between restriction sites EcoRI and XbaI are available in Table S4. gRNA[At] (sequence in Table S5) is encoded in plasmid pgRNA-humanized [Addgene plasmid #44248] as described by Lebar *et al.* (25)

### Cell culture and cultivation

**Human embryonic kidney (HEK) 293T** (ATTC) cell line was cultured in Dulbecco's modified Eagle's medium (DMEM) (Gibco) supplemented with 10% fetal bovine serum (Thermo Fisher Scientific) at 37°C and 5% CO_2_ and seeded at a density 2 × 10^4^ viable cells/well in Costar White clear bottom 96-well plates (Corning) for luciferase spectrometry experiments or 2 × 10^5^ viable cells/well in 6-well plate (TPP) for quantitative PCR experiments and flow cytometry.

**Mouse neuroblastoma Neuro2A** (ATTC) cell line was cultured in OptiMEM (Gibco) supplemented with 10% fetal bovine serum (Thermo Fisher Scientific) at 37°C and 5% CO_2_ and seeded at a density 5 × 10^4^ viable cells/well in Costar White clear bottom 96-well plates (Corning) for luciferase spectrometry experiments.

**Chinese Hamster ovary (CHO)** cell line was cultured in DMEM/F12 (Gibco) media, supplemented with 10% fetal bovine serum (Thermo Fisher Scientific) at 37°C and 5% CO_2_ and seeded at a density 2 × 10^4^ viable cells/well in Costar White clear bottom 96-well plates (Corning) for luciferase spectrometry experiments.

**HeLa** (ATCC) cell line was cultured in Dulbecco's modified Eagle's medium (DMEM) (Gibco) supplemented with 10% fetal bovine serum (Thermo Fisher Scientific) at 37°C and 5% CO_2_ and seeded at a density 1 × 10^4^ viable cells/well in Costar White clear bottom 96-well plates (Corning) for luciferase spectrometry experiments.

**Jurkat** (Invitrogen) cell line was cultured in RPMI (Gibco) media supplemented with 10% fetal bovine serum (Thermo Fisher Scientific) at 37°C and 5% CO_2_.

### Transient transfection

For luciferase assay measurements a plasmid with Renilla luciferase encoded under constitutive promoter, phRL-TK (Promega) was added to transfection mixtures indicated under individual figure as transfection control.

HEK 293T and HeLa cells were grown to 40–60% confluence and transfected with a mixture of jetPEI (Polyplus transfection) and DNA (3 ul/500 ng DNA, stock concentration 0.324 mg/ml, pH 7.5), a total of 225 ng plasmid DNA/well was transfected into cells.

Neuro2A and CHO cells were transfected with lipofectamine 2000 (Thermo Fischer Scientific) following the manufacturer's instructions immediately after seeding.

Jurkat cells were electroporated with Neon electroporation system (Thermo Fischer) in 100 μl tips at 1600 V, 10ms of pulse length and 3 pulses per sample. A total of 10 μg (for experiments with reporters with target sites for Gal4 and TetR) or 17.5 μg (for experiments with reporters with Zif268target sites) of DNA was used to electroporate 2 × 10^6^ cells for each sample. After electroporation, the cells were resuspended in 2 ml of fresh medium and seeded into 12-well plate.

All transfections were done in biological quadruplicates (HEK 293T, Neuro2A, CHO, HeLa) or technical quadruplicates (Jurkat) and all experiments were repeated at least three times.

For RNA-isolation cells were grown to 40–60% confluence. Transfection mixtures contained, where indicated on graph, 500 ng of A:tet reporter, 250 ng of vector encoding TetR:VP16 and 250 ng of vector encoding TALE[A] DNA-binding domain. DNA mixtures were combined with a mixture of jetPEI (Polyplus transfection; 3 ul/500 ng DNA, stock concentration 0.324 mg/ml, pH = 7,5) and transfected into HEK 293T cells. A total of 2600 ng plasmid DNA/well was transfected into cells.

For flow cytometry HEK 293T cells were grown to 40–60% confluence and transfected with a mixture of jetPEI (Polyplus transfection, 3 ul/500 ng DNA, stock concentration 0.324 mg/ml, pH 7.5) and DNA. Transfection mixtures contained, where indicated on graph, 800 ng of A:tet reporter, 400 ng of vector encoding TetR:VP16 and 400 ng of vector encoding TALE[A] DNA-binding domain. A total of 2600 ng plasmid DNA/well was transfected into cells.

### Luciferase assays

Cells were harvested 2 days after transfection, lysed with 1× passive lysis buffer (Promega), and a dual luciferase assay was performed using a Dual Luciferase Reporter Assay (Promega). Renilla and Firefly luminescence were measured on Orion II microplate reader (Berthold Technologies).

Relative luciferase units (RLU) were obtained by normalizing the firefly luciferase value to the *Renilla* luciferase value for each sample. All RLU were normalized to a reference value of reporter activity after addition of the transcription factor to obtain normalized RLU (nRLU) values.

Mean value and standard deviation of data were calculated and in GraphPad Prism 8 from 4 biological (CHO, HEK 293, HeLa, Neuro2A experiments) or technical (experiments on Jurkat) replicates. Each individual replicate is represented with a dot. Data was plotted on a graph in GraphPad Prism 8. Each experiment was repeated independently at least three times. Statistical significance between two groups of samples with and without TALE[A] was determined by unpaired two-tailed unequal variance *t*-test and the *P*-value is specified on a graph or in a Supplementary Table (* *P <* 0.05 ** *P <* 0.01 *** *P <* 0.001 NS: *P* > 0.1).

### RNA isolation, reverse transcription and quantitative PCR

Cells were harvested 2 days after transfection and washed with phosphate buffered saline. RNA was extracted using Purelink RNA mini kit (Invitrogen) following manufacturer's guidelines. DNA was digested by RQ1 RNase-Free DNase (Promega) and complementary (cDNA) was prepared by reverse transcription from 1 μg of RNA sample with random mix of RT reverse primers (Applied Biosystems) using a High-Capacity cDNA Reverse Transcription Kit (Applied Biosystems). Reaction was performed in 20 μl according to manufacturer's instructions (25 °C, 10 min; 37 °C, 120 min; 85 °C, 5 min; 10 °C, 10 min)

The obtained cDNA was diluted 10× and 5 μl of cDNA was added to each reaction mix. A qPCR was performed using LightCycler^®^ 480 SYBR Green I Master kit (Roche) on LightCycler 480 Instrument (Roche). Reporter transcription was detected by luc2CP specific primers (5’-TCGTGCTGGAACACGGTAAA-3’ and 5’-AACTTGCCGGTCAGTCCTTT-3’) and hygromycin specific primers (5’-TCGTCTGCGAGCCTACATGC-3’ and 5’-TCGAAGTTGCCGTCCACGAG-3’) as internal control to account for transfection efficiency. All primers were diluted to final concentration 400 nM. Reaction was performed under the following conditions: 95°C, 15 min; 45× (95 °C, 15 s; 60 °C, 30 s; 72 °C, 30 s), 37 °C, 10 s. To ensure qPCR specificity a melting curve analysis was performed on LightCycler®480 Instrument. Quantification analysis was performed on LightCycler®480 Instrument with LightCycler®480 Software release 1.5.1.62 and cycle of quantification (Cq) values were derived by second derivative maximum (SDM) method. Average value from three technical replicates was normalized to the rate of transcription after addition of an activator TetR:VP16 (control). The results are displayed as luc2CP relative to hygromycin gene expression and presented as the fold increase relative control and were calculated using the 2^−ΔΔCT^ formula (26).

Mean value and standard deviation were calculated in GraphPad Prism 8 from three independent experiments. Data was plotted on a graph in GraphPad Prism 8. Error bars represent the standard deviation of data. Each individual replicate is represented with a dot. Statistical significance between samples with and without TALE[A] was determined by unpaired two-tailed unequal variance *t*-test and the *P*-value is specified on a graph (* *P <* 0.05, ** *P <* 0.01, *** *P <* 0.001, NS: *P* > 0.1).

### Flow cytometry

Two days after transfection cells were resuspended in 500 μl of the media. Flow cytometry was performed on Aurora flow cytometer (Cytek). A 405-diode laser was used for BFP excitation. In each sample, 40 000 cells were analyzed and gated to singlets. The data was processed and presented on graph using Flow-Jo 10 software (TreeStar). Experiments were repeated independently at least three times.

### 3D molecular models

Molecular models of double-stranded B-DNA were generated by a web tool ‘DNA sequence to structure (http://www.scfbio-iitd.res.in/software/drugdesign/bdna.jsp#1)’ based on DNA sequence, including binding sites for both DNA-binding proteins. For modelling Gal4 and Zif268 PDB structures 1QPI, 3COQ and 1AAY were used, respectively. As the model of TetR bound to DNA was not available, a 1QPI structure was used. For TALE[A] a model of a TALE[A] described by Lebar *et al.* (23,27) was used. The alignment of the amino acid sequences encoded on the vector and presented by the model was made using the Needleman-Wunsch algorithm by EMBL-EBI’s Pairwise sequence alignment tool (https://www.ebi.ac.uk/Tools/psa/emboss_needle/). All the models were combined in Chimera by comparing and aligning the proteins to dsDNA using Chimera's MatchMaker functionality (28). To check for steric clash between proteins on dsDNA a clash analyzing tool build into UCSF Chimera was used (28).

Molecular models of Zif268 with transcriptional activator VP16 (Zif268:VP16) were constructed using MODELLER (29). For VP16 a partial structure resolved by NMR was used (PDB ID: 2K2U). As a 3D structure of full VP16 is not available and likely contains disordered regions, two modeling strategies were used: (i) only Zif268 or (ii) both Zif268 and partial VP16 structures were used as reference structures. Protein regions without reference structures were treated as unstructured. 150 models were generated for each strategy using custom scripts. All the models were combined in Chimera by comparing and aligning the proteins to dsDNA using Chimera's MatchMaker functionality (28). To check for clashes between proteins on dsDNA a clash analyzing tool build into UCSF Chimera was used (28). Details of in silico analysis are listed in Table S6, computational models are available in Supplementary Model. A representative Zif268:VP16 model was selected and visualized.

### Figures

All Figures were prepared in Inkscape (https://inkscape.org).

## RESULTS

**Figure 1.**
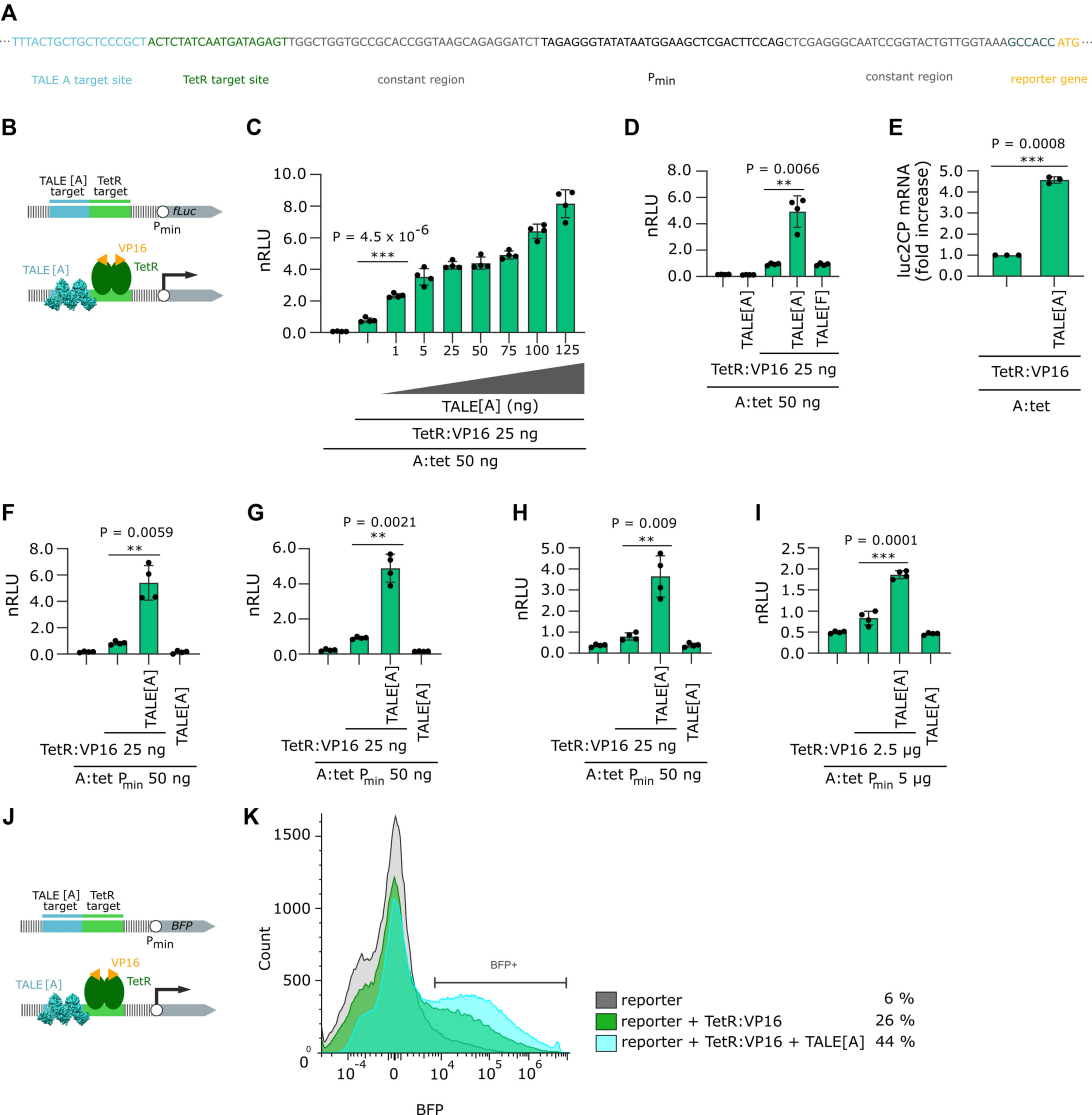
Analysis of TALE[A] effect on reporter transcription and activity. (**A**) Nucleotide sequence of the reporter plasmid, upstream of reporter gene. All the parts changed throughout this study are annotated, the rest of the sequence remains constant. For full annotation see supplement. (**B**) Schematic representation of the luciferase reporter used in experiments C-I with (bottom) and without (top) depiction of TALE[A] and TetR:VP16 bound to their target sites. (**C**) Measurement of luciferase activity corresponding to increasing amounts of TALE[A] encoding plasmid transfected. The experiment was performed on HEK 293T cell line. (**D**) Measurement of luciferase activity on HEK 293T cell line. TALE[F] is a negative control for TALE binding to target DNA as the reporter has no binding site for TALE[F]. Where indicated, 25 ng of TALE-encoding plasmids were co-transfected. (**E**) Quantitative RT-PCR of luc2CP mRNA. Values are normalized to TetR:VP16 activation. The bars represent the mean ± s.d.; *n* = 3 separate experiments. Transfection mixtures of plasmids were performed as indicated in qPCR methods. Experiment was performed on HEK 293T cell line. (**F**) Measurement of luciferase activity on Neuro2A cell line. Where indicated, 25 ng of TALE-encoding plasmids were co-transfected. (**G**) Measurement of luciferase activity on CHO cell line. Where indicated, 25 ng of TALE-encoding plasmids were co-transfected. (**H**) Measurement of luciferase activity on HeLa cell line. Where indicated, 25 ng of TALE-encoding plasmids were co-transfected. (**I**) Measurement of luciferase activity on Jurkat cell line. Where indicated, 2.5 μg of TALE-encoding plasmids were co-electroporated. The bars represent the mean ± s.d.; *n* = 4 technical replicates. (**J**) Schematic representation of the BFP reporter used in experiment K with (bottom) and without (top) depiction of TALE[A] and TetR:VP16 bound to their targets. (**K**) Flow cytometry histogram. Percentage of BFP positive singlets is indicated next to legend. HEK 293T cells were gated to singlets and plotted by their BFP fluorescence. Transfection mixtures were performed as stated in cytometry methods. All relative luciferase units were normalized (nRLU) to luciferase activity elicited by TetR:VP16. The bars represent the mean ± s.d.; *n* = 4 biologically independent cell cultures, unless stated otherwise. Statistical significance between samples with an activator and with and without TALE[A] was determined by unpaired two-tailed unequal variance *t*-test and the *P*-value is specified on graph. Transfection mixtures of plasmids were performed as indicated below graph, unless stated otherwise.

Through examination of the positional effect of TALE on transcription in mammalian cells driven by a transcriptional activator, we observed that binding of TALEs in close proximity of a transcriptional activator affected upregulation of the reporter introduced into a mammalian cell line HEK 293T. To further investigate the observed effect, a series of constructs were prepared with a defined separation between binding sites, their orientations and types of DNA-binding domains. The parts of the sequence altered during this study are annotated in Figure 1A, the rest of the sequence upstream of reporter gene remains constant.

### TALE binding enhances transcriptional activity

Reporters were named as a combination of two target sites, so that the reporter with a target site for TALE[A] (hereafter referred to as A) and a target site for TetR:VP16, a fusion protein of TetR DNA-binding domain and VP16 activation domain, was named A:tet. The reporter plasmid A:tet was co-transfected with a vector encoding the transcription factor TetR:VP16 and an increasing amount of a vector encoding the DNA-binding domain of TALE[A] protein, lacking C-terminal activation domain (Figure 1B). As shown in Figure 1C, the addition of as little as 1 ng of the TALE[A] plasmid to the transfection mixture containing the A:tet reporter and the vector encoding TetR:VP16 resulted in significant increase of the reporter activity. The effect depended on the TALE[A] concentration, as the reporter activity was more pronounced with an increasing amount of co-transfected TALE[A] encoding plasmid and enhanced transcriptional activity 3- to 8-fold compared to the activation elicited by TetR:VP16 alone.

TALE[A] alone had no effect on transcriptional activation in the absence of TetR:VP16, suggesting a synergistic action of both DNA-binding proteins when bound in the proximity (Figure 1D). We wanted to confirm that TALE causes this effect by binding to its respective site and not by interacting with another protein in the transcription machinery. To test this was not the case, TALE[F], which had no target site on the plasmid, was used instead of TALE[A]. As shown by the data presented in Figure 1D, the presence of a TALE without its binding site had no effect, confirming that the effect was due to the binding of a TALE protein to its respective DNA target in the proximity of the TetR binding site.

To determine whether the observed effect of the TALE[A] affects the amount of reporter mRNA transcript, quantitative RT-PCR was performed. Figure 1E confirms this assumption and shows that binding of the TALE[A] to a reporter adjacently to TetR:VP16 target site increases transcriptional activation more than 4-fold.

Furthermore, we show that the effect of transcriptional enhancement can be observed in different cell lines (Figure 1F-I). The same plasmids as in the experiments shown in Figure 1D and 1E were introduced into different cell lines. The results in the Neuro2A (Figure 1F) and CHO (Figure 1G) cell lines show a similar effect to the HEK 293T cell line, namely a 4- to 6-fold upregulation, whereas TALE[A] by itself showed no effect. In the HeLa (Figure 1H) and Jurkat (Figure 1I) cell lines, the transcriptional activity achieved by TetR:VP16 was barely above background, which may be due to lower transfection or electroporation efficiency (for HeLa and Jurkat, respectively). Nevertheless, in these two cell lines, the effect of TALE[A] upregulation was clearly evident.

Additionally, we investigated the effect at the single-cell level using a BFP reporter (Figure 1J) to demonstrate that this effect is not reporter-specific. The ratio of reporter, activator, and TALE[A]-encoding plasmids was preserved (2:1:1, respectively). Flow cytometry results in Figure 1K show an increase in cell population with high BFP fluorescence, confirming that the effect is not just a side-effect of a bulk measurement, but can be observed at the level of a single cell.

### Different TALE proteins and orientation maintain the transcription enhancement while other DNA-binding proteins do not exhibit the same effect.

**Figure 2.**
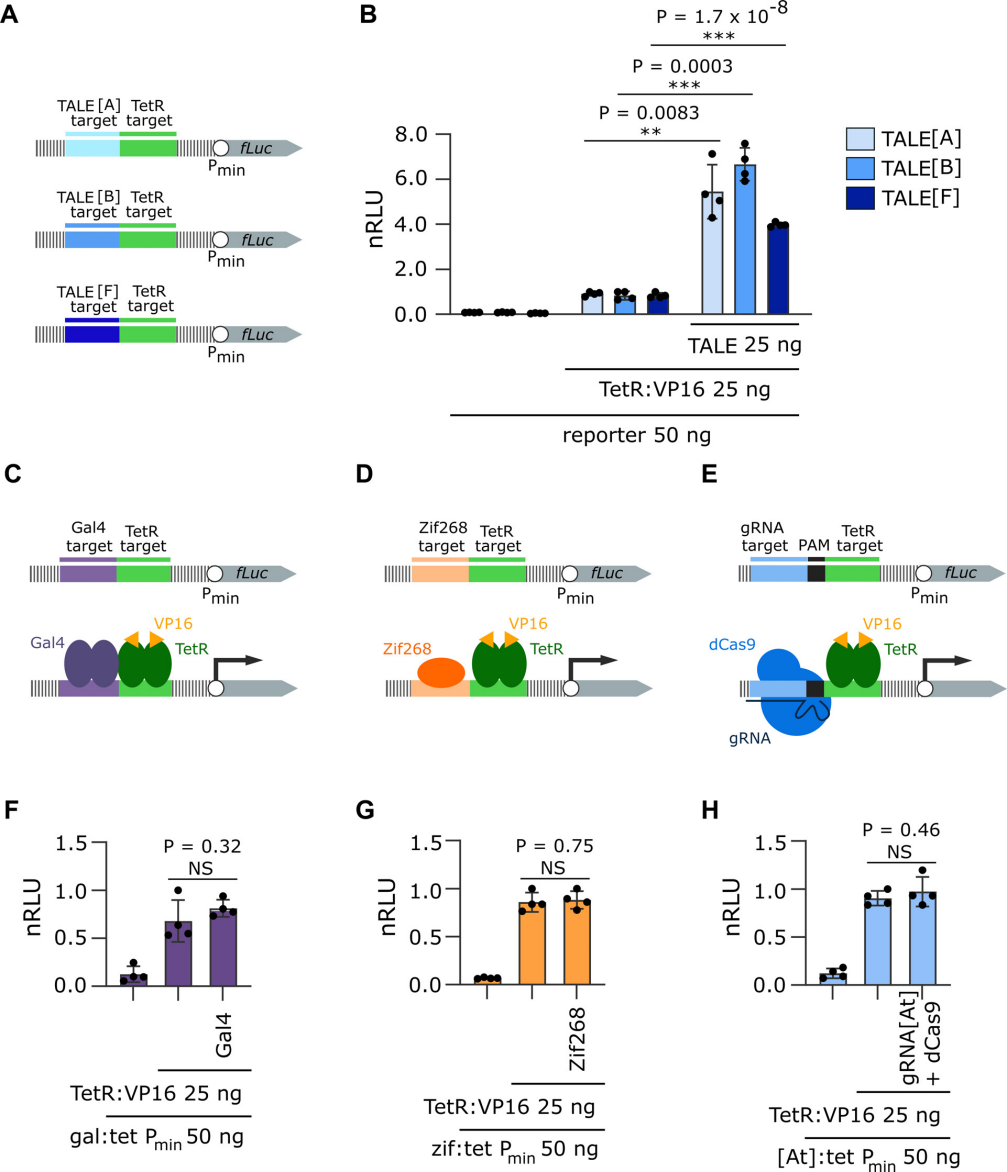
Effect of TALE and several other DNA-binding proteins on transcriptional activation. (**A**) Schematic representation of the reporters used in experiment B (**B**) Measurement of luciferase activity elicited by different TALE proteins binding to their correspondent binding site. (**C**) Schematic representation of the reporters used in experiment F (**D**) Schematic representation of the reporters used in experiment G. (**E**) Schematic representation of the reporters used in experiment H. (**F**) Measurement of luciferase activity. The added amount of Gal4-encoding plasmid is 25 ng. (**G**) Measurement of luciferase activity. The added amount of Zif268-encoding plasmid is 100 ng. (**H**) Measurement of luciferase activity. The added amount of gDNA[At] and dCas9-encoding plasmid is 50 ng each. All experiments were performed on HEK 293T cell line. Relative luciferase units were normalized (nRLU) to luciferase activity elicited by TetR:VP16. The bars represent the mean ± s.d.; n = 4 biologically independent cell cultures. Statistical significance between samples with an activator with and without TALE[A] was determined by unpaired two-tailed unequal variance t-test and the p-value is specified on graph. Transfection mixtures of plasmids were performed as indicated below each graph.

To confirm that the effect is a feature of TALE proteins and not just this particular variant, an experiment was designed with upstream target sites for several different TALE proteins. Reporters were constructed with target sites for three different TALE proteins, namely TALE[A], TALE[B] and TALE[F], whose target sites were labelled A, B, and F, respectively (Figure 2A). The experiment confirmed an enhancement of transcriptional activation for all tested TALE proteins (Figure 2B). An important question is whether this enhancement effect might also be exhibited by other DNA-binding proteins that bind DNA in a different manner. To test this, reporters with upstream target sites for three other types of DNA-binding proteins without effector domains were designed (Gal4, Zif268 and dCas9; Figure 2C–E, respectively). The presence of neither Gal4, Zif268 or dCas9/gRNA altered the transcriptional activation elicited by TetR:VP16 (Figure 2F–H, respectively), suggesting that the observed enhancement effect is a feature of TALE protein binding and not a general effect of DNA-binding proteins. Combined, these results show TALE proteins have a unique ability to enhance the effect of a neighboring transcription factor.

**Figure 3.**
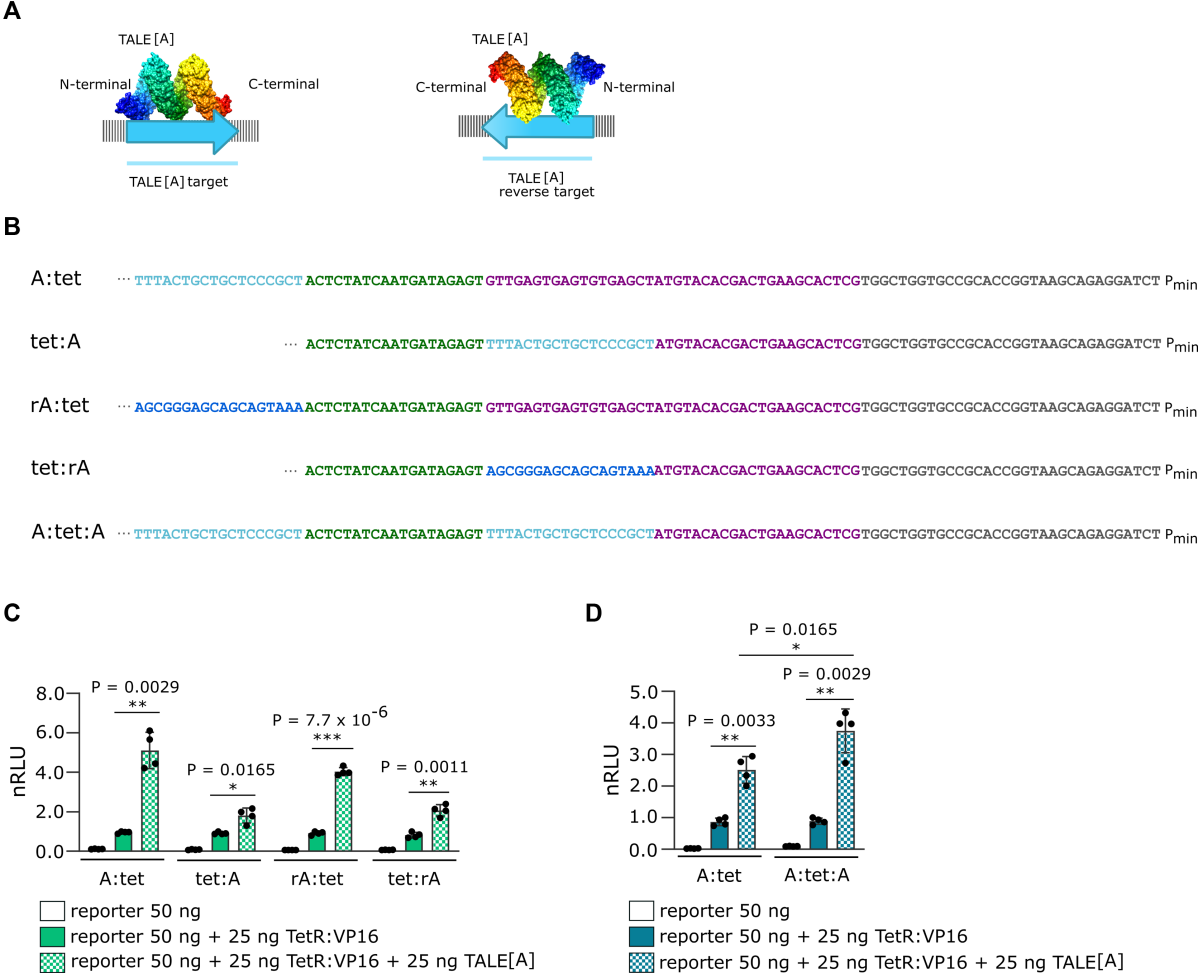
Analysis of transcriptional activation affected by different TALE protein binding orientations and positions. (**A**) Schematic representation of the designed reverse TALE[A] binding site with bound proteins colored to illustrate the chain from the N terminal (blue) to the C terminal (red). rA is the reverse complement of the TALE[A] target site. (**B**) Nucleotide sequences of the reporter plasmids used in experiments C and D, upstream of promotor. Light blue = TALE[A] target site, dark blue = TALE[A] reverse target site, green = TetR target site, gray = constant region, magenta = spacer to ensure distance of transcription factor target site to promoter is the same for all constructs. For full annotation see supplement. (**C**) Measurement of luciferase activity examining the effect of different placements and orientations of the TALE[A] target site adjacently to TetR target site on the enhancement of transcriptional activation. Statistical significance between samples with an activator with and without TALE[A] was determined by unpaired two-tailed unequal variance t-test and the p-value is specified on graph. (**D**) Measurement of luciferase activity examining the effect TALE[A] with target sites only upstream or both upstream and downstream of the TetR:VP16 target site. Statistical significance between samples with an activator with and without TALE[A] and between samples of reporters A:tet and A:tet:A with an activator and TALE[A] was determined by unpaired two-tailed unequal variance *t*-test and the p-value is specified on graph. All experiments were performed on HEK 293T cell line. Transfection mixtures of plasmids were performed as indicated in each legend. Relative luciferase units were normalized (nRLU) to luciferase activity elicited by TetR:VP16. The bars represent the mean ± s.d.; *n* = 4 biologically independent cell cultures unless stated otherwise.

To further characterize the TALE-mediated enhancement of transcriptional activation, we designed plasmids with varying placements of the TALE[A] target site relative to the other transcription factor binding site. The TALE[A] target site was placed on either 5’ or 3’ side of the activator target site in both original (A) or reverse complement (rA) sequence (Figure 3A) to investigate both orientations of TALEs bound to DNA; the resulting plasmids were labelled A:tet, tet:A, rA:tet, and tet:rA (Figure 3B). While the effect is most pronounced when TALE[A] is bound to the 5’ side of TetR:VP16, it also resulted in the increased transcriptional activation, when TALE[A] was bound in the reverse orientation (Figure 3C). On the other hand, when bound to the 3’ side of TetR:VP16, the effect was still present, however to a lesser extent, which may be due to its position relative to the promoter. This suggests that the observed increase in transcriptional activation is not caused by a direct interaction between TALE[A] and TetR:VP16.

One possible mechanism might be that TALE[A] facilitates TetR:VP16’s search for its binding site along the DNA chain. If this is the case, TALE[A] bound to both sides of the TetR target site could hinder TetR:VP16 binding. Reporters to test this possibility were constructed (Figure 3B). While binding of TALE[A] to either the 5’ or the 3’ side of the TetR target site resulted in transcriptional enhancement in both cases (Figure 3C), binding of TALE[A] to both the 5’ and the 3’ sides actually enhanced transcription slightly more than when binding to the 5’ side alone (Figure 3D).

**Figure 4.**
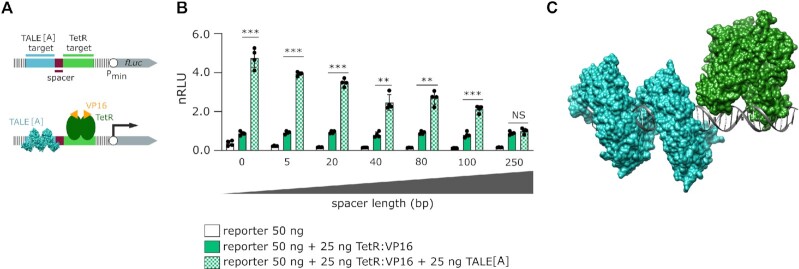
The effect of distance between target sites on TALE[A] elicited transcriptional activation (**A**) A schematic representation of the reporters used in this experiment with (bottom) and without (top) proteins bound. The length of DNA altered in this experiment is indicated in magenta. (**B**) Measurement of luciferase activity. Transfection mixtures of plasmids were performed as indicated in the legend. The experiment was performed on HEK 293T cell line. Relative luciferase units were normalized (nRLU) to luciferase activity elicited by TetR:VP16. The bars represent the mean ± s.d.; *n* = 4 biologically independent cell cultures. Statistical significance between samples with an activator and with and without TALE[A] was determined by unpaired two-tailed unequal variance *t*-test and the corresponding *P*-values are listed in Table S8: * *P <*0.05 ** *P <*0.01 *** *P <*0.001, NS: *P*> 0.1. (**C**) The 3D model of TALE[A] (cyan) and TetR (green) bound to their respective target sites.

Determining that direct contact between TALE[A] and TetR:VP16 likely is not necessary for the effect, we wanted to test the effect of the distance between DNA target sites (Figure 4A). All sequences were tested with GPMiner (30) to ensure that they did not contain binding sites for endogenous mammalian transcription factors and promoter regions. While TALE[A] bound directly upstream of TetR:VP16 elicited the highest activation increase, the effect slowly decreased with the increasing distance of the TALE target site from the TetR target site, as shown in Figure 4B (the corresponding *P*-values are listed in Table S8). Remarkably, the effect persisted even when the target sites were separated by as much as 100 bp, which still retained more than 2-fold increase. This further refutes the idea of a direct interaction between TetR:VP16 and TALE[A] proteins. Inspection of a 3D model (Figure 4C and Supplementary Video 1) of both proteins bound to their adjacent target sites reveals no contact between TALE[A] and TetR, prompting the question whether the TALE[A]-elicited enhancement of transcriptional activation is specific to TetR:VP16 or whether it might be observed in combination with other transcription factors.

### Synergistic effect of TALE with diverse transcription factors

**Figure 5.**
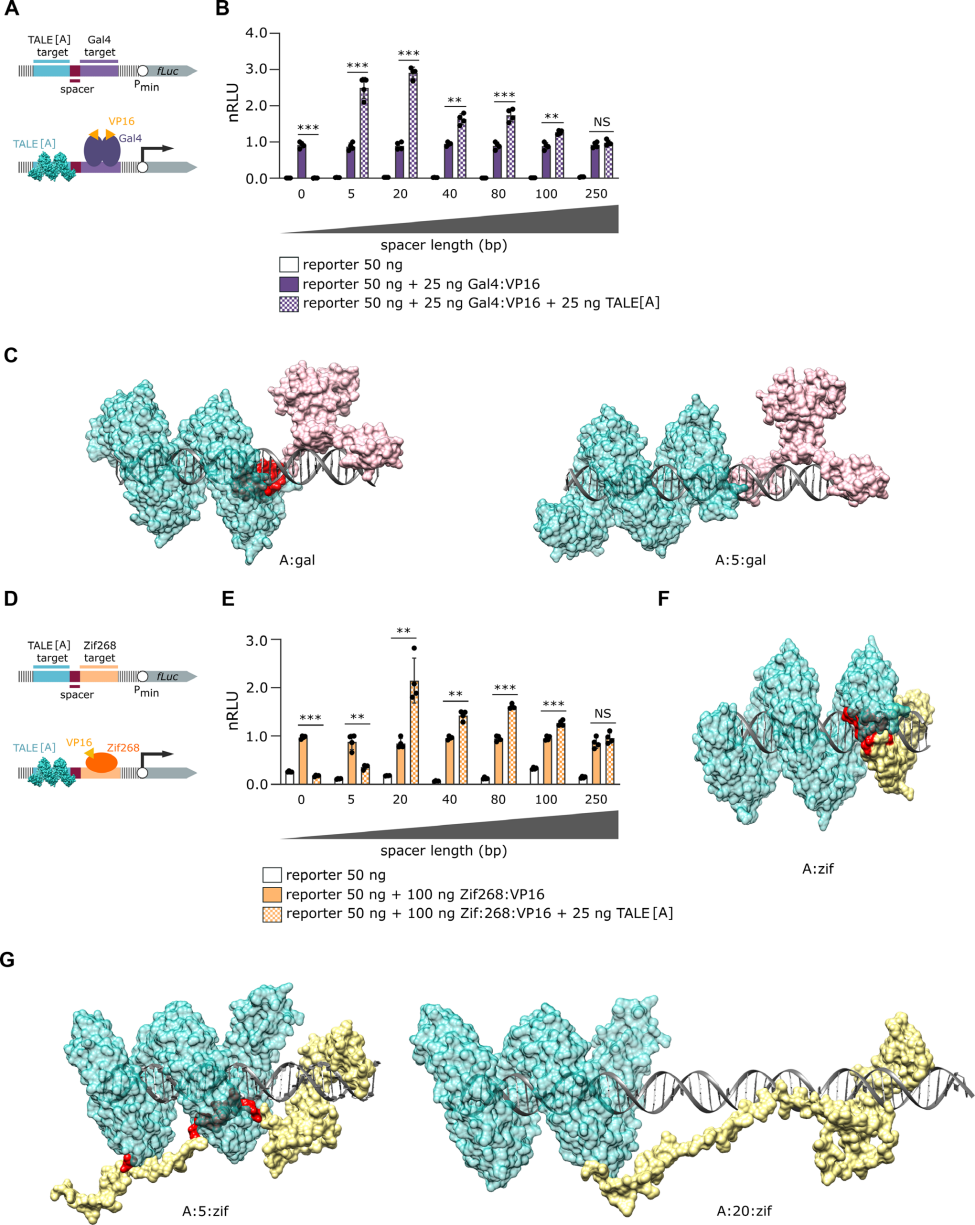
Effect of TALE on transcriptional activation in combination with different types of transcription factors. (**A**) Schematic representation of the reporters used in experiment B, with (bottom) and without (top) proteins bound. The length of DNA altered in this experiment is indicated in magenta. (**B**) Measurement of luciferase activity elicited by the effect of TALE[A] on Gal4:VP16. (**C**) 3D model of the TALE[A] dsDNA Gal4 complex shown in cyan-grey-pink, respectively. The red surface represents a steric obstruction. The TALE[A] surface is partially transparent, so the red surface is better visible. Sequence labels and spacers are indicated below each model. (**D**) Schematic representation of the reporters used in experiment E, with (bottom) and without (top) bound proteins. The length of DNA altered in this experiment is indicated in magenta. (**E**) Measurement of luciferase activity elicited by the effect of TALE[A] on Zif268:VP16. (**F**) Model of TALE[A] dsDNA Zif268 complex shown in cyan-grey-yellow, respectively. The red surface represents the steric obstruction. The TALE[A] surface is partially transparent, so the red surface is better visible. (**G**) Model of the TALE[A] dsDNA Zif268:VP16 complex shown in cyan-grey-yellow, respectively. The red surface represents the steric obstruction. The TALE[A] surface is partially transparent, so the red surface is better visible. Sequence labels and spacers are indicated below each model. All experiments were performed on HEK 293T cell line. Transfection mixtures of plasmids were performed as indicated in legends. Relative luciferase units were normalized (nRLU) to luciferase activity elicited by activator. The bars represent the mean ± s.d.; *n* = 4 biologically independent cell cultures. Statistical significance between samples with and without TALE[A] was determined by unpaired two-tailed unequal variance t-test and the corresponding p-values are listed in Table S9 and S10 for B and E, respectively. * *P <*0.05, ** *P <*0.01, *** *P <*0.001, NS: *P*> 0.1.

Target sites for a TALE protein and different types of DNA-binding domains of designed transcription factors were placed upstream of a minimal promoter controlling a firefly luciferase reporter gene (Figure 5A, 5D). Transcription factors investigated were constructed by fusing a DNA-binding domain of Gal4 or Zif268 (with target sites gal and zif, respectively) to the VP16 activation domain. These transcription factors differ in fold and DNA-binding mode: in Gal4 DNA-binding domain two helices of a dimer interact with the DNA, while the zinc finger Zif268 interacts with a major groove of DNA as a monomer. We demonstrated previously (23) that TALE proteins are able to displace the transcription factor bound to its 3’ side when there is a steric overlap between a TALE and another DNA-bound protein. Indeed, in case of both Gal4 and Zif268 the repression of transcription was observed due to the displacement in case of a juxtaposed binding sites (no spacer) (Figure 5B, 5E; the corresponding p-values are listed in Table S9 and S10; respectively). When the target sites of TALE[A] and Gal4 were separated by a 5 bp spacer, the TALE-induced transcriptional enhancement was already apparent, although it was still weaker than in the case of TetR, most likely due to the combination of the opposing effects of the displacement and enhancement. The transcription amplification even increased when the distance between the binding sites was extended to 20 bp and then gradually decreased, whereas for TetR the highest enhancement was observed when the binding sites of TALE[A] and TetR were adjacent to each other. Similarly, for Zif268:VP16, the addition of a 5 bp spacer between target sites resulted in a partial release of inhibition due to displacement, but introduction of a 20 bp spacer significantly enhanced transcriptional activation (Figure 5E), demonstrating that TALE-induced enhancement of transcriptional activation occurs for diverse types of transcription factors.

*In silico* modelling of proteins bound to DNA without spacers between target sites revealed a spatial clash of the TALE protein and the adjacent transcription factor (Figure 5C and Supplementary Video 2 for Gal4 and Figure 5F and Supplementary Video 4 for Zif268), visualizing the displacement effect observed in Figure 5B and 5E. A spacer of 5 bp introduced sufficient distance between TALE[A] and the Gal4 target sites to decrease the displacement and the transcription enhancement feature of TALE became apparent (Figure 5C and Supplementary Video 3). In the case of Zif268:VP16 model, steric hindrance persisted for a 5 bp spacer but was eliminated when the spacer length was increased to 20 bp (Figure 5G and Supplementary Videos 5 and 6). The reduced steric hindrance in these models is consistent with the results in Figure 5E. Model of TALE[A] has been previously described (23) and represents most of the encoded protein (Alignment S1). All the above models incorporated DNA-binding domain of TALEs lacking most of the N- and C-terminal ends that are unstructured.

**Figure 6.**
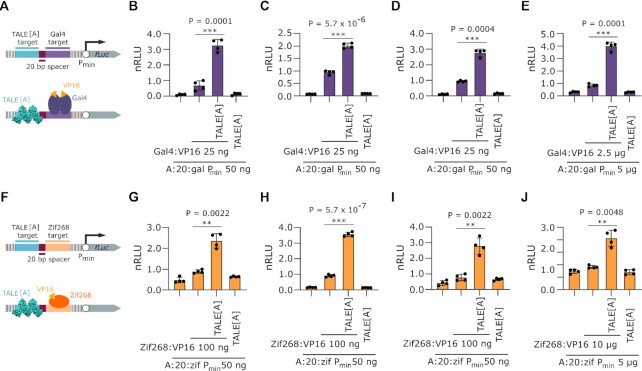
Effect of TALE on transcriptional activation in different cell lines. (**A**) Schematic representation of the luciferase reporter used in experiments B-E with (bottom) and without (top) depiction of TALE[A] and Gal4:VP16 bound to their targets. (**B**) Measurement of luciferase activity on Neuro2A cell line. Where indicated, 25 ng of TALE-encoding plasmids were co transfected. (**C**) Measurement of luciferase activity on CHO cell line. Where indicated, 25 ng of TALE-encoding plasmids were co transfected. (**D**) Measurement of luciferase activity on HeLa cell line. Where indicated, 25 ng of TALE-encoding plasmids were co transfected. (**E**) Measurement of luciferase activity on Jurkat cell line. Where indicated, 2.5 μg of TALE-encoding plasmids were co-electroporated. The bars represent the mean ± s.d.; *n* = 4 technical replicates. (**F**) Schematic representation of the luciferase reporter used in experiments G-J with (bottom) and without (top) depiction of TALE[A] and Zif268:VP16 bound to their targets. (**G**) Measurement of luciferase activity on Neuro2A cell line. Where indicated, 25 ng of TALE-encoding plasmids were co transfected. (H) Measurement of luciferase activity on CHO cell line. Where indicated, 25 ng of TALE-encoding plasmids were co transfected. (**I**) Measurement of luciferase activity on HeLa cell line. Where indicated, 25 ng of TALE-encoding plasmids were co transfected. (**J**) Measurement of luciferase activity on Jurkat cell line. Where indicated, 2.5 μg of TALE-encoding plasmids were co-electroporated. The bars represent the mean ± s.d.; *n* = 4 technical replicates. All transfection mixtures of plasmids were performed as indicated below graph. Relative luciferase units were normalized (nRLU) to luciferase activity elicited by an activator. The bars represent the mean ± s.d.; *n* = 4 biologically independent cell cultures, unless stated otherwise. Statistical significance between samples with and without TALE[A] was determined by unpaired two-tailed unequal variance *t*-test and the *P*-value is specified on graph.

In order to demonstrate that the observed effect is not unique to the HEK 293T cell line, we performed similar experiments in several other cell lines. The reporters (Figure 6A and 6F for Gal4 and Zif268, respectively) and the plasmid ratio used were those that resulted in the most pronounced effects in previous experiments (Figure 5B and 5E for Gal4 and Zif268, respectively). The results for all four cell lines (Figure 6B and 6G for N2A, Figure 6C and 6H for CHO, Figure 6D and 6I for HeLa, and Figure 6E and 6J for Jurkat cell line) confirm the robustness of the TALE[A] induced transcriptional enhancement also for these two transcription factors as well.

These results show TALEs can act cooperatively in combination with several types of transcription factors. This was demonstrated for homodimeric transcription factors such as TetR:VP16 and Gal4:VP16 as well as for a monomeric transcription factor such as Zif268:VP16.

### Binding of TALEs to a proximal site can augment transcriptional repression

**Figure 7.**
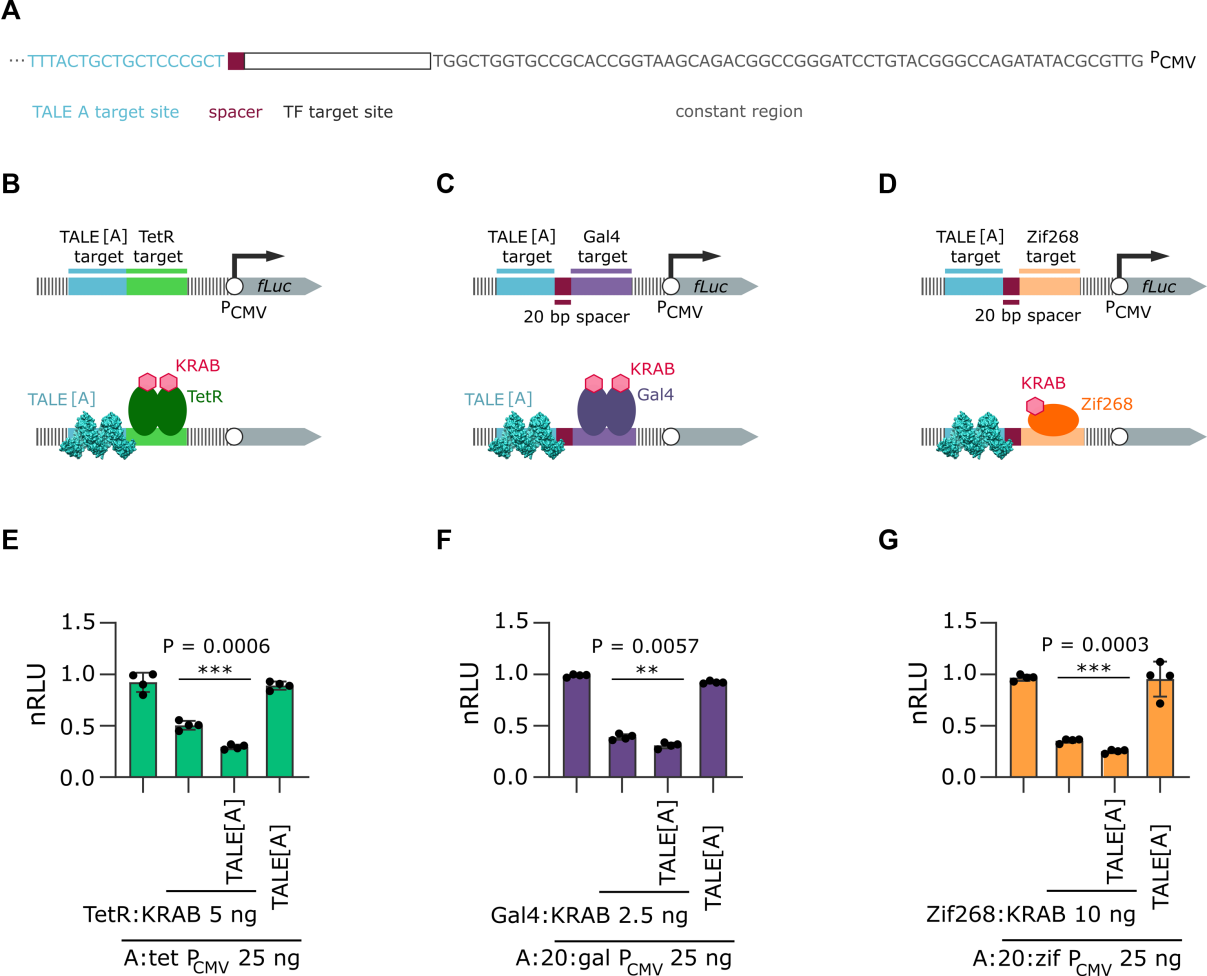
Effect of TALE on augmentation of transcriptional repression. (**A**) Nucleotide sequence of the reporter plasmid, upstream of reporter gene. All the parts changed throughout this experiment are annotated, the rest of the sequence remains constant. For full annotation see supplement. (**B**) Schematic representation of the reporters used in experiment E. with (bottom) and without (top) proteins bound. (**C**) Schematic representation of the reporters used in experiment F. with (bottom) and without (top) proteins bound. (**D**) Schematic representation of the reporters used in experiment G. with (bottom) and without (top) proteins bound. (**E**) Measurement of luciferase activity elicited by the effect of TALE[A] on TetR:KRAB. (**F**) Measurement of luciferase activity elicited by the effect of TALE[A] on Gal4:KRAB. (**G**) Measurement of luciferase activity elicited by the effect of TALE[A] on Zif268:VP16. All experiments were performed on HEK 293T cell line. Transfection mixtures of plasmids were performed as indicated in legends, where indicated, 25 ng of TALE[A] encoding plasmid was co transfected. Relative luciferase units were normalized (nRLU) to luciferase activity elicited by reporter alone. The bars represent the mean ± s.d.; *n* = 4 biologically independent cell cultures. Statistical significance between samples with and without TALE[A] was determined by unpaired two-tailed unequal variance t-test and the *P*-value is specified on graph.

We wondered whether the cooperative effect of TALEs on transcriptional activators might also occur with transcriptional repressors. This would be expected if the effect were due to potentiation of another transcriptional regulator. The effect of TALE binding was therefore investigated in a repression system comprising combination of reporter plasmids containing target sites for a TALE protein and KRAB-containing repressor placed upstream of a strong pCMV promoter controlling a firefly luciferase reporter gene (Figure 7A). The distance between the target sites for a TALE protein and a transcriptional repressor was adjusted to the distance that resulted in the most pronounced effect in previous experiments, as shown in Figure 4B, Figure 5B, and 5E for TetR, Gal4, and Zif268, respectively (Figure 7B–D). The transcription factors investigated were constructed by fusing a DNA-binding domain of TetR, Gal4, or Zif268 (with target sites tet, gal, and zif, respectively) to a KRAB repression domain.

Indeed, augmented repression was observed, when TALE[A] and TetR:KRAB, Gal4:KRAB, or Zif268:KRAB were used (Figure 7E, 7F and 7G, respectively), whereas the TALE[A] plasmid co-transfected with the reporter plasmid alone had no effect. This suggests that the presence of the TALE protein augments activity of a nearby transcription factor, regardless whether it is an activator or a repressor.

## DISCUSSION

The discovered ability of TALE proteins to enhance transcriptional regulation of other transcription factors further expands the variety of the effects of TALE proteins on transcription, which could act as an additional modulator in regulating transcriptional pathways.

TALE does not appear to require direct interaction with transcription factors because orientation, upstream or downstream position, and spacing between binding sites, as well as different types of DNA-binding domains, maintain this effect, albeit with some variability to the distance and position. This suggests that DNA, the connecting ligand, may be able to convey the binding event of a TALE that improves binding characteristics at the transcription factor target site. The effect was maintained regardless of the homodimeric or monomeric target transcription factor.

Over the last decades several research groups have described cooperative actions that could not be explained otherwise than by DNA intervention, and various allosteric mechanisms have been proposed (8,9,11). In addition to the strengthening of weaker protein-protein interactions by DNA, known as the direct readout mechanism, which can be ruled out because the effect was maintained over the distance of tens of base pairs, other allosteric mechanisms have been proposed: The indirect readout mechanism, in which binding of the primary protein bends the DNA and improves binding properties of the secondary binding site, the solvent release mechanism, in which changes in water or ion distribution caused by primary binding are thought to reduce the desolvation cost, required for the binding of the second protein, and entropy-mediated cooperativity, in which primary binding alters the vibrational modes of DNA and generates a perturbation wave that propagates across DNA to distal regions (8,9).

TALE wraps around the DNA in the form of a superhelix, and the crystal structures available in the literature (17,31) show no strong bending of the target DNA, so the indirect readout mechanism is not likely. The ability of the TALE protein to cooperatively interact with transcription factors over distances up to 100 bp rules out the solvent release mechanism, as its effect should be more local (10,32). We observed that TALE[A] acts synergistically with diverse transcription factors regardless of orientation and the effect can span 100 bp, which is consistent with DNA- mediated allostery that exhibits low specificity for a DNA-binding partner and can act over a range of tens of base pairs, which can’t be achieved by other described modes of cooperativity (8,9). TALEs could represent a pioneering transcription factor (33) that could release DNA to enable more efficient binding of other transcription factors. Recent publications described cooperative protein binding without distortion of DNA structure that spans multiple nucleotides between binding sites (9–11,34).

Several processes in nature are mediated by altering protein expression and, because transcription in eukaryotes occurs through cooperative and synergistic actions of an ensemble of proteins, allosteric proteins have interesting potential as tools for synthetic biology (12). While we have demonstrated the effect of TALE proteins on ectopically expressed DNA-binding proteins, it is possible that the observed effect of TALEs may also be demonstrated by some natural transcription factors. TALEs have been found in pathogenic bacteria that subvert the immune response in plants and have been primarily attributed to the activation domain of bacterial TALEs; however, the contribution of the DNA-binding domain of TALEs on other DNA-binding proteins remains to be seen. In addition to expanding our understanding of the variety of synergistic effects on transcriptional regulation, a designable DNA-binding protein with characterized cooperative properties could serve as a tool to investigate the mechanisms of synergistic transcriptional regulation and harvest its potential.

## DATA AVAILABILITY

All relevant data is included in the main manuscript and the supplementary material.

## Supplementary Material

gkac454_Supplemental_Files

## References

[1] Perez-Pinera P. , Ousterout D.G., Brunger J.M., Farin A.M., Glass K.A., Guilak F., Crawford G.E., Hartemink A.J., Gersbach C.A. Synergistic and tunable human gene activation by combinations of synthetic transcription factors. Nat. Methods. 2013; 10:239–242. 23377379 10.1038/nmeth.2361 PMC3719416

[2] Gerstein M.B. , Kundaje A., Hariharan M., Landt S.G., Yan K.K., Cheng C., Mu X.J., Khurana E., Rozowsky J., Alexander R. et al. Architecture of the human regulatory network derived from ENCODE data. Nature. 2012; 489:91–100. 22955619 10.1038/nature11245 PMC4154057

[3] Yan J. , Enge M., Whitington T., Dave K., Liu J., Sur I., Schmierer B., Jolma A., Kivioja T., Taipale M. et al. Transcription factor binding in human cells occurs in dense clusters formed around cohesin anchor sites. Cell. 2013; 154:801–813. 23953112 10.1016/j.cell.2013.07.034

[4] Lambert S.A. , Jolma A., Campitelli L.F., Das P.K., Yin Y., Albu M., Chen X., Taipale J., Hughes T.R., Weirauch M.T. The human transcription factors. Cell. 2018; 172:650–665. 29425488 10.1016/j.cell.2018.01.029 PMC12908702

[5] Jolma A. , Yin Y., Nitta K.R., Dave K., Popov A., Taipale M., Enge M., Kivioja T., Morgunova E., Taipale J. DNA-dependent formation of transcription factor pairs alters their binding specificity. Nature. 2015; 527:384–388. 26550823 10.1038/nature15518

[6] Williams D.C. , Cai M., Clore G.M. Molecular basis for synergistic transcriptional activation by oct1 and sox2 revealed from the solution structure of the 42-kDa oct1·sox2· Hoxb1-DNA ternary transcription factor complex. J. Biol. Chem. 2004; 279:1449–1457. 14559893 10.1074/jbc.M309790200

[7] Merabet S. , Saadaoui M., Sambrani N., Hudry B., Pradel J., Affolter M., Graba Y. A unique extradenticle recruitment mode in the drosophila hox protein ultrabithorax. Proc. Natl. Acad. Sci. U.S.A. 2007; 104:16946–16951. 17942685 10.1073/pnas.0705832104 PMC2040397

[8] Morgunova E. , Taipale J. Structural perspective of cooperative transcription factor binding. Curr. Opin. Struct. Biol. 2017; 47:1–8. 28349863 10.1016/j.sbi.2017.03.006

[9] Balaceanu A. , Pérez A., Dans P.D., Orozco M. Allosterism and signal transfer in DNA. Nucleic Acids Res. 2018; 46:7554–7565. 29905860 10.1093/nar/gky549 PMC6125689

[10] Talavera A. , Tamman H., Ainelo A., Konijnenberg A., Hadži S., Sobott F., Garcia-Pino A., Hõrak R., Loris R. A dual role in regulation and toxicity for the disordered N-terminus of the toxin graT. Nat. Commun. 2019; 10:972. 30814507 10.1038/s41467-019-08865-z PMC6393540

[11] Kim S. , Broströmer E., Xing D., Jin J., Chong S., Ge H., Wang S., Gu C., Yang L., Gao Y.Q. et al. Probing allostery through DNA. Science. 2013; 339:816–819. 23413354 10.1126/science.1229223 PMC3586787

[12] Raman S. , Taylor N., Genuth N., Fields S., Church G.M. Engineering allostery. Trends Genet. 2014; 30:521–528. 25306102 10.1016/j.tig.2014.09.004 PMC4254034

[13] De Lange O. , Binder A., Lahaye T. From dead leaf, to new life: TAL effectors as tools for synthetic biology. Plant J. 2014; 78:753–771. 24602153 10.1111/tpj.12431

[14] Cuculis L. , Abil Z., Zhao H., Schroeder C.M. Direct observation of TALE protein dynamics reveals a two-state search mechanism. Nat. Commun. 2015; 6:7277. 26027871 10.1038/ncomms8277 PMC4458887

[15] Cuculis L. , Abil Z., Zhao H., Schroeder C.M. TALE proteins search DNA using a rotationally decoupled mechanism. Nat. Chem. Biol. 2016; 12:831–837. 27526029 10.1038/nchembio.2152

[16] Deng D. , Yan C., Wu J., Pan X., Yan N. Revisiting the TALE repeat. Protein Cell. 2014; 5:297–306. 24622844 10.1007/s13238-014-0035-2 PMC3978159

[17] Mak A.N.S. , Bradley P., Cernadas R.A., Bogdanove A.J., Stoddard B.L. The crystal structure of TAL effector pthxo1 bound to its DNA target. Science. 2012; 335:716–719. 22223736 10.1126/science.1216211 PMC3427646

[18] Deng D. , Yan C., Pan X., Mahfouz M., Wang J., Zhu J.K., Shi Y., Yan N. Structural basis for sequence-specific recognition of DNA by TAL effectors. Science (80-.). 2012; 335:720–723. 10.1126/science.1215670 PMC3586824 22223738

[19] Rinaldi F.C. , Doyle L.A., Stoddard B.L., Bogdanove A.J. The effect of increasing numbers of repeats on TAL effector DNA binding specificity. Nucleic Acids Res. 2017; 45:6960–6970. 28460076 10.1093/nar/gkx342 PMC5499867

[20] Miller J.C. , Tan S., Qiao G., Barlow K.A., Wang J., Xia D.F., Meng X., Paschon D.E., Leung E., Hinkley S.J. et al. A TALE nuclease architecture for efficient genome editing. Nat. Biotechnol. 2011; 29:143–150. 21179091 10.1038/nbt.1755

[21] Li Y. , Moore R., Guinn M., Bleris L. Transcription activator-like effector hybrids for conditional control and rewiring of chromosomal transgene expression. Sci. Rep. 2012; 2:897. 23193439 10.1038/srep00897 PMC3508452

[22] Werner J. , Gossen M. Modes of TAL effector-mediated repression. Nucleic Acids Res. 2014; 42:13061–13073. 25389273 10.1093/nar/gku1124 PMC4245958

[23] Lebar T. , Verbič A., Ljubetič A., Jerala R. Polarized displacement by transcription activator-like effectors for regulatory circuits. Nat. Chem. Biol. 2019; 15:80–87. 30455466 10.1038/s41589-018-0163-8

[24] Gibson D.G. , Young L., Chuang R.Y., Venter J.C., Hutchison C.A., Smith H.O. Enzymatic assembly of DNA molecules up to several hundred kilobases. Nat. Methods. 2009; 6:343–345. 19363495 10.1038/nmeth.1318

[25] Lebar T. , Lainšček D., Merljak E., Aupič J., Jerala R. A tunable orthogonal coiled-coil interaction toolbox for engineering mammalian cells. Nat. Chem. Biol. 2020; 16:513–519. 31907374 10.1038/s41589-019-0443-y PMC7182445

[26] Livak K.J. , Schmittgen T.D. Analysis of relative gene expression data using real-time quantitative PCR and the 2−ΔΔCT method. Methods. 2001; 25:402–408. 11846609 10.1006/meth.2001.1262

[27] Sander J.D. , Cade L., Khayter C., Reyon D., Peterson R.T., Joung J.K., Yeh J.R.J. Targeted gene disruption in somatic zebrafish cells using engineered TALENs. Nat. Biotechnol. 2011; 29:697–698. 21822241 10.1038/nbt.1934 PMC3154023

[28] Pettersen E.F. , Goddard T.D., Huang C.C., Couch G.S., Greenblatt D.M., Meng E.C., Ferrin T.E. UCSF chimera - a visualization system for exploratory research and analysis. J. Comput. Chem. 2004; 25:1605–1612. 15264254 10.1002/jcc.20084

[29] Webb B. , Sali A. Comparative protein structure modeling using MODELLER. Curr. Protoc. Bioinforma. 2016; 2016:5.6.1–5.6.37. 10.1002/cpbi.3 PMC5031415 27322406

[30] Lee T.Y. , Chang W.C., Hsu J.B.K., Chang T.H., Shien D.M. GPMiner: an integrated system for mining combinatorial cis-regulatory elements in mammalian gene group. Biomed Central Genomics. 2012; 13:Imperial College Press S3. 10.1186/1471-2164-13-S1-S3 PMC3587379 22369687

[31] Gao H. , Wu X., Chai J., Han Z. Crystal structure of a TALE protein reveals an extended N-terminal DNA binding region. Cell Res. 2012; 22:1716–1720. 23147789 10.1038/cr.2012.156 PMC3515758

[32] Harris L.A. , Williams L.D., Koudelka G.B. Specific minor groove solvation is a crucial determinant of DNA binding site recognition. Nucleic Acids Res. 2014; 42:14053–14059. 25429976 10.1093/nar/gku1259 PMC4267663

[33] Zaret K.S. Pioneer transcription factors initiating gene network changes. Annu. Rev. Genet. 2020; 54:367–385. 32886547 10.1146/annurev-genet-030220-015007 PMC7900943

[34] Rosenblum G. , Elad N., Rozenberg H., Wiggers F., Jungwirth J., Hofmann H. Allostery through DNA drives phenotype switching. Nat. Commun. 2021; 12:2967. 34016970 10.1038/s41467-021-23148-2 PMC8170675

