# Supplementary material for: Binding of the transcription activator-like effector augments transcriptional regulation by another transcription factor
Source: Nucleic Acids Res. 2022 Jun 7;50(11):6562–74. doi: 10.1093/nar/gkac454 (PMC9226504; doi:10.1093/nar/gkac454)
Supplement: gkac454_Supplemental_Files [file gkac454_supplemental_files.zip › Leben_Supplementary_material_REVISED.pdf]

Binding of the Transcription Activator-Like Effector augments transcriptional regulation by another transcription factor

Katja Leben[1, 2], Žiga Strmšek[1], Tina Lebar[1], Anže Verbič[1, 2], Matej Dragovan[1], Neža Omersa[1], Gregor Anderluh[1], Roman Jerala[1]*

[1] Department of Synthetic Biology and Immunology, National Institute of Chemistry, Hajdrihova 19, SI-1000 Ljubljana, Slovenia

[2] Interdisciplinary Doctoral Programme in Biomedicine, University of Ljubljana, Kongresni trg 12, SI-1000 Ljubljana, Slovenia

* To whom correspondence should be addressed. Tel: +386 1 476 035; Email: roman.jerala@ki.si

List of Alignments in Supplementary materials:

Alignment 1: Alignment of amino acid sequences encoded by the reporter plasmid used in the experiments (TALE[A] plasmid) and amino acid sequence of the model (TALE[A] model)

List of Tables in Supplementary materials:

Table S1: Origin of DNA sequences used in cloning in this study
Table S2: DNA sequences of transcriptional activation reporters used in this study. The sequences specified are positioned between 'synthetic poly (A) signal/ transcriptional pause site' and 'luc2CP' feature of pGL4.16 vector.
Table S3: DNA sequences of transcriptional repression reporters used in this study. The sequences specified are positioned between AmpR promoter feature and CMV promoter in pcDNA3 with luc2CP cloned between HindII and XbaI restriction sites, as described in the methods.
Table S4: Amino acid sequences of proteins used in this study. Proteins were encoded between EcoRI and XbaI restriction sites of pcDNA3 vector.
Table S5: RNA seqence used in this study
Table S6: : *In silico* analysis of TALE[A] and Zif:VP16 on indicated reporters
Table S7: : Reporter sequences other than luc2CP used in this study. Sequence was introduced to pGL4.16 vector right after sequence A:tet (Table S2).
Table S8: P-values corresponding to statistical analysis of samples with tet reporter and TetR:VP16 in Figure 4B with and without added TALE[A].
Table S9: P-values corresponding to statistical analysis of samples with gal reporter and Gal4:VP16 in Figure 5B with and without added TALE[A].
Table S10: P-values corresponding to statistical analysis of samples with zif reporter and Zif268:VP16 in Figure 5E with and without added TALE[A].

List of supplementary videos

Supplementary video 1: A movie of a 3D model of TALE[A] and TetR bound to their respective target sites shown in cyan and green, respectively. DNA is shown in gray. The video was recorded in Chimera.

Supplementary video 2: A movie of a 3D model of TALE[A] and Gal4 bound to their respective target sites on DNA shown in cyan and pink, respectively. The red surface represents a steric obstruction. The TALE[A] surface is partially transparent, so the red surface is better visible. The video was recorded in Chimera.

Supplementary video 3: A movie of a 3D model of TALE[A] and Gal4 bound to their respective target sites separated by 5 bp shown in cyan and pink, respectively. DNA is shown in gray. The TALE[A] surface is partially transparent. The video was recorded in Chimera.

Supplementary video 4: A movie of a 3D model of TALE[A] and Zif268 bound to their respective target sites shown in cyan and yellow, respectively. DNA is shown in gray. The red surface represents a steric obstruction. The TALE[A] surface is partially transparent, so the red surface is better visible. The video was recorded in Chimera.

Supplementary video 5: A movie of a 3D model of TALE[A] and Zif268:VP16 bound to their respective target sites separated by 5 bp shown in cyan and yellow, respectively. DNA is shown in gray. The red surface represents a steric obstruction. The TALE[A] surface is partially transparent, so the red surface is better visible. The video was recorded in Chimera.

Supplementary video 6: A movie of a 3D model of TALE[A] and Zif268:VP16 bound to their respective target sites separated by 20 bp shown in cyan and yellow, respectively. DNA is shown in gray. The TALE[A] surface is partially transparent. The video was recorded in Chimera.

List of supplementary models

Supplementary model 1: An overlay of all 300 generated models of Zif268:VP16, indicating possible conformations that the protein could be present in. Models were constructed using Modeller and homology modelling, using Zif268 and VP16 partial structures as a reference for Modeller.

Alignment S1: Alignment of amino acid sequences encoded by the reporter plasmid used in the experiments (TALE[A] plasmid) and amino acid sequence of the model (TALE[A] model)

```
TALE[A]plasmid  1 MHHHHHHDYKDHDGDYKDHDIDYKDDDDKMAPKKKRKVGIHRGVPMVDLR     50
                                                              ||||
TALE[A] model   1 ----------------------------------------------VDLR      4

TALE[A]plasmid 51 TLGYSQQQQEKIKPKVRSTVAQHHEALVGHGFTHAHIVALSQHPAALGTV    100
                  ||||||||||||||||||||||||||||||||||||||||||||||||||
TALE[A] model   5 TLGYSQQQQEKIKPKVRSTVAQHHEALVGHGFTHAHIVALSQHPAALGTV     54

TALE[A]plasmid101 AVKYQDMIAALPEATHEAIVGVGKQWSGARALEALLTVAGELRGPPLQLD    150
                  ||||||||||||||||||||||||||||||||||||||||||||||||||
TALE[A] model  55 AVKYQDMIAALPEATHEAIVGVGKQWSGARALEALLTVAGELRGPPLQLD    104

TALE[A]plasmid151 TGQLLKIAKRGGVTAVEAVHAWRNALTGAPLNLTPDQVVAIASNGGGKQA    200
                  ||||||||||||||||||||||||||||||||||||||||||||||||||
TALE[A] model 105 TGQLLKIAKRGGVTAVEAVHAWRNALTGAPLNLTPDQVVAIASNGGGKQA    154

TALE[A]plasmid201 LETVQRLLPVLCQDHGLTPEQVVAIASNGGGKQALETVQRLLPVLCQAHG    250
                  ||||||||||||||||||||||||||||||||||||||||||||||||||
TALE[A] model 155 LETVQRLLPVLCQDHGLTPEQVVAIASNGGGKQALETVQRLLPVLCQAHG    204

TALE[A]plasmid251 LTPDQVVAIASNIGGKQALETVQRLLPVLCQAHGLTPAQVVAIASHDGGK    300
                  ||||||||||||||||||||||||||||||||||||||||||||||||||
TALE[A] model 205 LTPDQVVAIASNIGGKQALETVQRLLPVLCQAHGLTPAQVVAIASHDGGK    254

TALE[A]plasmid301 QALETVQRLLPVLCQDHGLTPDQVVAIASNGGGKQALETVQRLLPVLCQD    350
                  ||||||||||||||||||||||||||||||||||||||||||||||||||
TALE[A] model 255 QALETVQRLLPVLCQDHGLTPDQVVAIASNGGGKQALETVQRLLPVLCQD    304

TALE[A]plasmid351 HGLTPEQVVAIANNNGGKQALETVQRLLPVLCQAHGLTPDQVVAIASHDG    400
                  ||||||||||||||||||||||||||||||||||||||||||||||||||
TALE[A] model 305 HGLTPEQVVAIANNNGGKQALETVQRLLPVLCQAHGLTPDQVVAIASHDG    354

TALE[A]plasmid401 GKQALETVQRLLPVLCQAHGLTPAQVVAIASNGGGKQALETVQRLLPVLC    450
                  ||||||||||||||||||||||||||||||||||||||||||||||||||
TALE[A] model 355 GKQALETVQRLLPVLCQAHGLTPAQVVAIASNGGGKQALETVQRLLPVLC    404

TALE[A]plasmid451 QDHGLTPDQVVAIANNNGGKQALETVQRLLPVLCQDHGLTPEQVVAIASH    500
                  ||||||||||||||||||||||||||||||||||||||||||||||||||
TALE[A] model 405 QDHGLTPDQVVAIANNNGGKQALETVQRLLPVLCQDHGLTPEQVVAIASH    454

TALE[A]plasmid501 DGGKQALETVQRLLPVLCQAHGLTPDQVVAIASNGGGKQALETVQRLLPV    550
                  ||||||||||||||||||||||||||||||||||||||||||||||||||
TALE[A] model 455 DGGKQALETVQRLLPVLCQAHGLTPDQVVAIASNGGGKQALETVQRLLPV    504

TALE[A]plasmid551 LCQAHGLTPAQVVAIASHDGGKQALETVQRLLPVLCQDHGLTPDQVVAIA    600
                  ||||||||||||||||||||||||||||||||||||||||||||||||||
TALE[A] model 505 LCQAHGLTPAQVVAIASHDGGKQALETVQRLLPVLCQDHGLTPDQVVAIA    554

TALE[A]plasmid601 SHDGGKQALETVQRLLPVLCQDHGLTPEQVVAIASHDGGKQALETVQRLL    650
                  ||||||||||||||||||||||||||||||||||||||||||||||||||
TALE[A] model 555 SHDGGKQALETVQRLLPVLCQDHGLTPEQVVAIASHDGGKQALETVQRLL    604

TALE[A]plasmid651 PVLCQAHGLTPDQVVAIANNNGGKQALETVQRLLPVLCQAHGLTPAQVVA    700
                  ||||||||||||||||||||||||||||||||||||||||||||||||||
TALE[A] model 605 PVLCQAHGLTPDQVVAIANNNGGKQALETVQRLLPVLCQAHGLTPAQVVA    654

TALE[A]plasmid701 IASHDGGKQALETVQRLLPVLCQDHGLTPEQVVAIASNGGGRPALESIVA    750
                  ||||||||||||||||||||||||||||||||||||||||||||||||||
TALE[A] model 655 IASHDGGKQALETVQRLLPVLCQDHGLTPEQVVAIASNGGGRPALESIVA    704

TALE[A]plasmid751 QLSRPDPALAALTNDHLVALACLGGRPALDAVKKGLPHAPALIKRTNRRI    800
                  ||||||||||||||||||||||||||||||||||||||||||||||||||
TALE[A] model 705 QLSRPDPALAALTNDHLVALACLGGRPALDAVKKGLPHAPALIKRTNRRI    754

TALE[A]plasmid801 PERTSHRVAGSDPKKKRKV    819
                  |||||||||||
TALE[A] model 755 PERTSHRVAGS--------    765
```

Table S1: Origin of DNA sequences used in this study

LABEL	ORIGIN
TALE[A]	PCR amplified from TALEN1257 (aa 2-756) [Addgene plasmid 32280]
TALE[B]	PCR amplified from TALEN1297 (aa 2-756) [Addgene plasmid 32279]
TALE[F]	PCR amplified from TAL 3006 (aa 2-756) [Addgene plasmid 35998]
TetR	PCR amplified from bioBrick Bba_C0040
Gal4	PCR amplified from Bind-Gal4VP16 provided by Iain M. Morgan (Institute of Comparative Medicine, Glasgow, United Kingdom)
Zif268	synthesized by LifeTechnologies
VP16	PCR amplified from from the vector pSGVP, provided by prof. Mark Ptashne (Memorial Sloan-Kettering Cancer Center, New York)
KRAB	PCR amplified from pLVPT-rtTR-KRAB-2SM2 [Addgene plasmid 11652]
SV40 nls	introduced into constructs with PCR
BFP	Cloned from pTagBFP-N (Evrogen)

Table S2: DNA sequences of transcriptional activation reporters used in this study. The sequences specified are positioned between 'synthetic poly (A) signal/ transcriptional pause site' and 'luc2CP' feature of pGL4.16 vector.

LABEL	SEQUENCE
A:tet	CTGCTTAGGGTTAGGCGTTTTGCGCTGCTTTTACTGCTGCTCCCGCTACTCTATCAATGATAGAGTTGGCTGGTGCCGCACCGGTAAGCAGAGGATCTTAGAGGGTATATAATGGAAGCTCGACTTCCAGCTCGAGGGCAATCCGGTACTGTTGGTAAAGCCACC<br>plasmid:TALE[A] target site:TetR target site:plasmid:Pmin:plasmid:Kozak's sequence
B:tet	CTGCTTAGGGTTAGGCGTTTTGCGCTGCTTCTTCCGTTTCCACATCTACTCTATCAATGATAGAGTTGGCTGGTGCCGCACCGGTAAGCAGAGGATCTTAGAGGGTATATAATGGAAGCTCGACTTCCAGCTCGAGGGCAATCCGGTACTGTTGGTAAAGCCACC<br>plasmid:TALE[B] target site:TetR target site:plasmid:Pmin:plasmid:Kozak's sequence
F:tet	CTGCTTAGGGTTAGGCGTTTTGCGCTGCTTCAATATGACCGTATGTGACTCTATCAATGATAGAGTTGGCTGGTGCCGCACCGGTAAGCAGAGGATCTTAGAGGGTATATAATGGAAGCTCGACTTCCAGCTCGAGGGCAATCCGGTACTGTTGGTAAAGCCACC<br>plasmid:TALE[F] target site:TetR target site:plasmid:Pmin:plasmid:Kozak's sequence
tet:A	CTGCTTAGGGTTAGGCGTTTTGCGCTGCTACTCTATCAATGATAGAGTTTTACTGCTGCTCCCGCTATGTACACGACTGAAGCACTCGTGGCTGGTGCCGCACCGGTAAGCAGAGGATCTTAGAGGGTATATAATGGAAGCTCGACTTCCAGCTCGAGGGCAATCCGGTACTGTTGGTAAAGCCACC<br>Plasmid:TetR target site:TALE[A] target site:plasmid:Pmin:plasmid:Kozak's sequence
rA:tet	CTGCTTAGGGTTAGGCGTTTTGCGCTGCTAGCGGGAGCAGCAGTAAAACTCTATCAATGATAGAGTGTTGAGTGAGTGTGAGCTATGTACACGACTGAAGCACTCGTGGCTGGTGCCGCACCGGTAAGCAGAGGATCTTAGAGGGTATATAATGGAAGCTCGACTTCCAGCTCGAGGGCAATCCGGTACTGTTGGTAAAGCCACC<br>plasmid:TALE[A] REVERSE target site:TetR target site:plasmid:Pmin:plasmid:Kozak's sequence
tet:rA	CTGCTTAGGGTTAGGCGTTTTGCGCTGCTACTCTATCAATGATAGAGTAGCGGGAGCAGCAGTAAAATGTACACGACTGAAGCACTCGTGGCTGGTGCCGCACCGGTAAGCAGAGGATCTTAGAGGGTATATAATGGAAGCTCGACTTCCAGCTCGAGGGCAATCCGGTACTGTTGGTAAAGCCACC<br>plasmid:TetR target site:TALE[A] REVERSE target site:plasmid:Pmin:plasmid:Kozak's sequence
A:tet (Figure 3C and E)	CTGCTTAGGGTTAGGCGTTTTGCGCTGCTTTTACTGCTGCTCCCGCTACTCTATCAATGATAGAGTGTTGAGTGAGTGTGAGCTATGTACACGACTGAAGCACTCGTGGCTGGTGCCGCACCGGTAAGCAGAGGATCTTAGAGGGTATATAATGGAAGCTCGACTTCCAGCTCGAGGGCAATCCGGTACTGTTGGTAAAGCCACC<br>plasmid:TALE[A] target site:TetR target site:plasmid:Pmin:plasmid:Kozak's sequence
A:tet:A	CTGCTTAGGGTTAGGCGTTTTGCGCTGCTTTTACTGCTGCTCCCGCTACTCTATCAATGATAGAGTTTTACTGCTGCTCCCGCTATGTACACGACTGAAGCACTCGTGGCTGGTGCCGCACCGGTAAGCAGAGGATCTTAGAGGGTATATAATGGAAGCTCGACTTCCAGCTCGAGGGCAATCCGGTACTGTTGGTAAAGCCACC<br>plasmid:TALE[A] target site:TetR target site:TALE[A] target site:plasmid:Pmin:plasmid:Kozak's sequence
A:5:tet	CTGCTTAGGGTTAGGCGTTTTGCGCTGCTTTTACTGCTGCTCCCGCTGTCAGACTCTATCAATGATAGAGTTGGCTGGTGCCGCACCGGTAAGCAGAGGATCTTAGAGGGTATATAATGGAAGCTCGACTTCCAGCTCGAGGGCAATCCGGTACTGTTGGTAAAGCCACC<br>plasmid:TALE[A] target site:5 bp spacer:TetR target site:plasmid:Pmin:plasmid:Kozak's sequence
A:20:tet	CTGCTTAGGGTTAGGCGTTTTGCGCTGCTTTTACTGCTGCTCCCGCTCAAAGACTTAAGAGTCTATCACTCTATCAATGATAGAGTTGGCTGGTGCCGCACCGGTAAGCAGAGGATCTTAGAGGGTATATAATGGAAGCTCGACTTCCAGCTCGAGGGCAATCCGGTACTGTTGGTAAAGCCACC<br>plasmid:TALE[A] target site:20 bp spacer:TetR target site:plasmid:Pmin:plasmid:Kozak's sequence
A:40:tet	CTGCTTAGGGTTAGGCGTTTTGCGCTGCTTTTACTGCTGCTCCCGCTGTTGAGTGAGTGTGAGCTATGTACACGACTGAAGCACTCGACTCTATCAATGATAGAGTTGGCTGGTGCCGCACCGGTAAGCAGAGGATCTTAGAGGGTATATAATGGAAGCTCGACTTCCAGCTCGAGGGCAATCCGGTACTGTTGGTAAAGCCACC<br>plasmid:TALE[A] target site:40 bp spacer:TetR target site:plasmid:Pmin:plasmid:Kozak's sequence
A:80:tet	CTGCTTAGGGTTAGGCGTTTTGCGCTGCTTTTACTGCTGCTCCCGCTGTAAGCTACGAACTCTTGCTTGAAGCTGTCTGCAGGAGAACCGCGTTGTTGACGTAGACTGCCTAGAGTCATGCTGACAGACTCTATCAATGATAGAGTTGGCTGGTGCCGCACCGGTAAGCAGAGGATCTTAGAGGGTATATAATGGAAGCTCGACTTCCAGCTCGAGGGCAATCCGGTACTGTTGGTAAAGCCACC<br>plasmid:TALE[A] target site:80 bp spacer:TetR target site:plasmid:Pmin:plasmid:Kozak's sequence
A:100:tet	CTGCTTAGGGTTAGGCGTTTTGCGCTGCTTTTACTGCTGCTCCCGCTGTTGAGTGAGTGTGAGCTATGTAAGCTACGAACTCTTGCTTGAAGCTGTCTGCAGGAGAACCGCGTTGTTGACGTAGACTGCCTAGAGTCATGCTGACAGACTCTATCAATGATAGAGTTGGCTGGTGCCGCACCGGTAAGCAGAGGATCTTAGAGGGTATATAATGGAAGCTCGACTTCCAGCTCGAGGGCAATCCGGTACTGTTGGTAAAGCCACC<br>plasmid:TALE[A] target site:100 bp spacer:TetR target site:plasmid:Pmin:plasmid:Kozak's sequence
A:250:tet	TTTTCAATATTATTGAAGCATTTATCAGGGTTTACTGCTGCTCCCGCTGGTTACTAGTACGTCTCTCAAGGATAAGTAAGTAATATTAAGGTACGGGAGGTATTGGACAGGCCGCAATAAAATATCTTTATTTTCATTACATCTGTGTGTTGGTTTTTTGTGTGAATCGATAGTACTAACATACGCTCTCCATCAAAACAAAACGAAACAAAACAAACTAGCAAAATAGGCTGTCCCCAGTGCAAGTGCAGGTGCCAGAACATTTCTCTCTGCTTAGGGTTAGGCGTTTTGCGCTGCTACTCTATCAATGATAGAGTTGGCTGGTGCCGCACCGGTAAGCAGAGGATCTTAGAGGGTATATAATGGAAGCTCGACTTCCAGCTCGAGGGCAATCCGGTACTGTTGGTAAAGCCACC<br>plasmid:TALE[A] target site:plasmid:SynPolyA/transcriptional stop:plasmid:TetR target site:plasmid:Pmin:plasmid:Kozak's sequence

Table S2 (continued)

LABEL	SEQUENCE
A:gal	CTGCTTAGGGTTAGGCGTTTTGCGCTGCTTTTACTGCTGCTCCCGCTCGGAGTACTGTCCTCCGTGGCTGGTGCCGCACCGGTAAGCAGAGGATCTTAGAGGGTATATAATGGAAGCTCGACTTCCAGCTCGAGGGCAATCCGGTACTGTTGGTAAAGCCACC<br>plasmid:TALE[A] target site:Gal4 target site:plasmid:Pmin:plasmid:Kozak's sequence
A:5:gal	CTGCTTAGGGTTAGGCGTTTTGCGCTGCTTTTACTGCTGCTCCCGCTGTCAGCGGAGTACTGTCCTCCGTGGCTGGTGCCGCACCGGTAAGCAGAGGATCTTAGAGGGTATATAATGGAAGCTCGACTTCCAGCTCGAGGGCAATCCGGTACTGTTGGTAAAGCCACC<br>plasmid:TALE[A] target site:5 bp spacer:Gal4 target site:plasmid:Pmin:plasmid:Kozak's sequence
A:20:gal	CTGCTTAGGGTTAGGCGTTTTGCGCTGCTTTTACTGCTGCTCCCGCTCAAAGACTTAAGAGTCTATCCGGAGTACTGTCCTCCGTGGCTGGTGCCGCACCGGTAAGCAGAGGATCTTAGAGGGTATATAATGGAAGCTCGACTTCCAGCTCGAGGGCAATCCGGTACTGTTGGTAAAGCCACC<br>plasmid:TALE[A] target site:20 bp spacer:Gal4 target site:plasmid:Pmin:plasmid:Kozak's sequence
A:40:gal	CTGCTTAGGGTTAGGCGTTTTGCGCTGCTTTTACTGCTGCTCCCGCTGTTGAGTGAGTGTGAGCTATGTACACGACTGAAGCACTCGCGGAGTACTGTCCTCCGTGGCTGGTGCCGCACCGGTAAGCAGAGGATCTTAGAGGGTATATAATGGAAGCTCGACTTCCAGCTCGAGGGCAATCCGGTACTGTTGGTAAAGCCACC<br>plasmid:TALE[A] target site:40 bp spacer:Gal4 target site:plasmid:Pmin:plasmid:Kozak's sequence
A:80:gal	CTGCTTAGGGTTAGGCGTTTTGCGCTGCTTTTACTGCTGCTCCCGCTGTAAGCTACGAACTCTTGCTTGAAGCTGTCTGCAGGAGAACCGCGTTGTTGACGTAGACTGCCTAGAGTCATGCTGACAGCGGAGTACTGTCCTCCGTGGCTGGTGCCGCACCGGTAAGCAGAGGATCTTAGAGGGTATATAATGGAAGCTCGACTTCCAGCTCGAGGGCAATCCGGTACTGTTGGTAAAGCCACC<br>plasmid:TALE[A] target site:80 bp spacer:Gal4 target site:plasmid:Pmin:plasmid:Kozak's sequence
A:100:gal	CTGCTTAGGGTTAGGCGTTTTGCGCTGCTTTTACTGCTGCTCCCGCTGTTGAGTGAGTGTGAGCTATGTAAGCTACGAACTCTTGCTTGAAGCTGTCTGCAGGAGAACCGCGTTGTTGACGTAGACTGCCTAGAGTCATGCTGACAGCGGAGTACTGTCCTCCGTGGCTGGTGCCGCACCGGTAAGCAGAGGATCTTAGAGGGTATATAATGGAAGCTCGACTTCCAGCTCGAGGGCAATCCGGTACTGTTGGTAAAGCCACC<br>plasmid:TALE[A] target site:100 bp spacer:Gal4 target site:plasmid:Pmin:plasmid:Kozak's sequence
A:250: gal	CTGCTTAGGGTTAGGCGTTTTGCGCTGCTTTTACTGCTGCTCCCGCTGGTTACTAGTACGTCTCTCAAGGATAAGTAAGTAATATTAAGGTACGGGAGGTATTGGACAGGCCGCAATAAAATATCTTTATTTTCATTACATCTGTGTGTTGGTTTTTTGTGTGAATCGATAGTACTAACATACGCTCTCCATCAAAACAAAACGAAACAAAACAAACTAGCAAAATAGGCTGTCCCCAGTGCAAGTGCAGGTGCCAGAACATTTCTCTCTGCTTAGGGTTAGGCGTTTTGCGCTGCTCGGAGTACTGTCCTCCGTGGCTGGTGCCGCACCGGTAAGCAGAGGATCTTAGAGGGTATATAATGGAAGCTCGACTTCCAGCTCGAGGGCAATCCGGTACTGTTGGTAAAGCCACC<br>plasmid:TALE[A] target site:plasmid:SynPolyA/transcriptional stop:plasmid:Gal4 target site:plasmid:Pmin:plasmid:Kozak's sequence
A:zif	CTGCTTAGGGTTAGGCGTTTTGCGCTGCTTTTACTGCTGCTCCCGCTGCGTGGGCGTTGGCTGGTGCCGCACCGGTAAGCAGAGGATCTTAGAGGGTATATAATGGAAGCTCGACTTCCAGCTCGAGGGCAATCCGGTACTGTTGGTAAAGCCACC<br>plasmid:TALE[A] target site:Zif268 target site:plasmid:Pmin:plasmid:Kozak's sequence
A:5:zif	CTGCTTAGGGTTAGGCGTTTTGCGCTGCTTTTACTGCTGCTCCCGCTGTCAGGCGTGGGCGTTGGCTGGTGCCGCACCGGTAAGCAGAGGATCTTAGAGGGTATATAATGGAAGCTCGACTTCCAGCTCGAGGGCAATCCGGTACTGTTGGTAAAGCCACC<br>plasmid:TALE[A] target site:5 bp spacer:Zif268 target site:plasmid:Pmin:plasmid:Kozak's sequence
A:20:zif	CTGCTTAGGGTTAGGCGTTTTGCGCTGCTTTTACTGCTGCTCCCGCTCAAAGACTTAAGAGTCTATCGCGTGGGCGTTGGCTGGTGCCGCACCGGTAAGCAGAGGATCTTAGAGGGTATATAATGGAAGCTCGACTTCCAGCTCGAGGGCAATCCGGTACTGTTGGTAAAGCCACC<br>plasmid:TALE[A] target site:20 bp spacer:Zif268 target site:plasmid:Pmin:plasmid:Kozak's sequence
A:40:zif	CTGCTTAGGGTTAGGCGTTTTGCGCTGCTTTTACTGCTGCTCCCGCTGTTGAGTGAGTGTGAGCTATGTACACGACTGAAGCACTCGGCGTGGGCGTTGGCTGGTGCCGCACCGGTAAGCAGAGGATCTTAGAGGGTATATAATGGAAGCTCGACTTCCAGCTCGAGGGCAATCCGGTACTGTTGGTAAAGCCACC<br>plasmid:TALE[A] target site:40 bp spacer:Zif268 target site:plasmid:Pmin:plasmid:Kozak's sequence
A:80:zif	CTGCTTAGGGTTAGGCGTTTTGCGCTGCTTTTACTGCTGCTCCCGCTGTAAGCTACGAACTCTTGCTTGAAGCTGTCTGCAGGAGAACCGCGTTGTTGACGTAGACTGCCTAGAGTCATGCTGACAGGCGTGGGCGTTGGCTGGTGCCGCACCGGTAAGCAGAGGATCTTAGAGGGTATATAATGGAAGCTCGACTTCCAGCTCGAGGGCAATCCGGTACTGTTGGTAAAGCCACC<br>plasmid:TALE[A] target site:80 bp spacer:Zif268 target site:plasmid:Pmin:plasmid:Kozak's sequence
A:100:zif	CTGCTTAGGGTTAGGCGTTTTGCGCTGCTTTTACTGCTGCTCCCGCTGTTGAGTGAGTGTGAGCTATGTAAGCTACGAACTCTTGCTTGAAGCTGTCTGCAGGAGAACCGCGTTGTTGACGTAGACTGCCTAGAGTCATGCTGACAGGCGTGGGCGTTGGCTGGTGCCGCACCGGTAAGCAGAGGATCTTAGAGGGTATATAATGGAAGCTCGACTTCCAGCTCGAGGGCAATCCGGTACTGTTGGTAAAGCCACC<br>plasmid:TALE[A] target site:100 bp spacer:Zif268 target site:plasmid:Pmin:plasmid:Kozak's sequence
A:250:zif	CTGCTTAGGGTTAGGCGTTTTGCGCTGCTTTTACTGCTGCTCCCGCTGGTTACTAGTACGTCTCTCAAGGATAAGTAAGTAATATTAAGGTACGGGAGGTATTGGACAGGCCGCAATAAAATATCTTTATTTTCATTACATCTGTGTGTTGGTTTTTTGTGTGAATCGATAGTACTAACATACGCTCTCCATCAAAACAAAACGAAACAAAACAAACTAGCAAAATAGGCTGTCCCCAGTGCAAGTGCAGGTGCCAGAACATTTCTCTCTGCTTAGGGTTAGGCGTTTTGCGCTGCTGCGTGGGCGTTGGCTGGTGCCGCACCGGTAAGCAGAGGATCTTAGAGGGTATATAATGGAAGCTCGACTTCCAGCTCGAGGGCAATCCGGTACTGTTGGTAAAGCCACC<br>plasmid:TALE[A] target site:plasmid:SynPolyA/transcriptional stop:plasmid:Zif268 target site:plasmid:Pmin:plasmid:Kozak's sequence
gal:tet	CTGCTTAGGGTTAGGCGTTTTGCGCTGCTCGGAGTACTGTCCTCCGACTCTATCAATGATAGAGTTGGCTGGTGCCGCACCGGTAAGCAGAGGATCTTAGAGGGTATATAATGGAAGCTCGACTTCCAGCTCGAGGGCAATCCGGTACTGTTGGTAAAGCCACC<br>plasmid:Gal4 target site:TetR target site:plasmid:Pmin:plasmid:Kozak's sequence
zif:tet	CTGCTTAGGGTTAGGCGTTTTGCGCTGCTGCGTGGGCGTACTCTATCAATGATAGAGTTGGCTGGTGCCGCACCGGTAAGCAGAGGATCTTAGAGGGTATATAATGGAAGCTCGACTTCCAGCTCGAGGGCAATCCGGTACTGTTGGTAAAGCCACC<br>plasmid: Zif268 target site:TetR target site:plasmid:Pmin:plasmid:Kozak's sequence
[At]:tet	CTGCTTAGGGTTAGGCGTTTTGCGCTGCTTTTACTGCTGCTCCCGCTACTCGGACTCTATCAATGATAGAGTTGGCTGGTGCCGCACCGGTAAGCAGAGGATCTTAGAGGGTATATAATGGAAGCTCGACTTCCAGCTCGAGGGCAATCCGGTACTGTTGGTAAAGCCACC<br>plasmid:gRNA binding site:PAM:TetR target site:plasmid:Pmin:plasmid:Kozak's sequence

Table S3: DNA sequences of transcriptional repression reporters used in this study. The sequences specified are positioned between AmpR promoter feature and CMV promoter in pcDNA3 with luc2CP cloned between HindII and XbaI restriction sites, as described in the methods.

LABEL	SEQUENCE
A:tet:Pcmv	TGTATTTAGAAAAATAAACAAATAGGGGTTCCGCGCACATTTCCCCGAAAAGTGCCACCTGACGTCGACGGATCG<br>GGAGATCTCCCGATCCCCTATGGTCGACTCTCAGTACAATCTGCTCTGATGCCGCATAGTTAAGCCAGTATCTGC<br>TCCCTGCTTGTGTGTTGGAGGTCGCTGAGTAGTGCGCGAGCAAAATTTAAGCTACAACAAGGCAAGGCTTGACCG<br>ACAATTGCATGAAGAATCTGCTTAGGGTTAGGCGTTTTGCGCTGCTTTTACTGCTGCTCCCGCTACTCTATCAAT<br>GATAGAGTTGGCTGGTGCCGCACCGGTAAGCAGACGGCCGGGATCCTGTACGGGCCAGATATACGCGTTG<br>Plasmid: TALE[A] target site: TetR target site:plasmid
A:20:gal:Pcmv	TGTATTTAGAAAAATAAACAAATAGGGGTTCCGCGCACATTTCCCCGAAAAGTGCCACCTGACGTCGACGGATCG<br>GGAGATCTCCCGATCCCCTATGGTCGACTCTCAGTACAATCTGCTCTGATGCCGCATAGTTAAGCCAGTATCTGC<br>TCCCTGCTTGTGTGTTGGAGGTCGCTGAGTAGTGCGCGAGCAAAATTTAAGCTACAACAAGGCAAGGCTTGACCG<br>ACAATTGCATGAAGAATCTGCTTAGGGTTAGGCGTTTTGCGCTGCTTTTACTGCTGCTCCCGCTCAAAGACTTAA<br>GAGTCTATCCGGAGTACTGTCCTCCGTGGCTGGTGCCGCACCGGTAAGCAGACGGCCGGGATCCTGTACGGGCCA<br>GATATACGCGTTG<br>Plasmid: TALE[A] target site:20 bp spacer:Gal4 target site:plasmid
A:20:zif:Pcmv	TGTATTTAGAAAAATAAACAAATAGGGGTTCCGCGCACATTTCCCCGAAAAGTGCCACCTGACGTCGACGGATCG<br>GGAGATCTCCCGATCCCCTATGGTCGACTCTCAGTACAATCTGCTCTGATGCCGCATAGTTAAGCCAGTATCTGC<br>TCCCTGCTTGTGTGTTGGAGGTCGCTGAGTAGTGCGCGAGCAAAATTTAAGCTACAACAAGGCAAGGCTTGACCG<br>ACAATTGCATGAAGAATCTGCTTAGGGTTAGGCGTTTTGCGCTGCTTTTACTGCTGCTCCCGCTCAAAGACTTAA<br>GAGTCTATCGCGTGGGCGTTGGCTGGTGCCGCACCGGTAAGCAGACGGCCGGGATCCTGTACGGGCCAGATATAC<br>GCGTTG<br>Plasmid: TALE[A] target site:20 bp spacer:Zif268 target site:plasmid

Table S4: Amino acid sequences of proteins used in this study. Proteins were encoded between EcoRI and XbaI restriction sites of pcDNA3 vector.

**LABEL**	**SEQUENCE**
TALE[A]	MHHHHHHDYKDHDGDYKDHDIDYKDDDDKMAPKKKRKVGIHRGVPMVDLRTLGYSQQQEKIKPKVRSTVAQHHE<br>ALVGHGFTHAHIVALSQHPAALGTVAVKYQDMIAALPEATHEAIVGVGKQWSGARALEALLTVAGELRGPPLQLD<br>TGQLLKIAKRGGVTAVEAVHAWRNALTGAPLNLTPDQVVAIASNGGGKQALETVQRLLPVLCQDHGLTPEQVVAI<br>ASNGGGKQALETVQRLLPVLCQAHGLTPDQVVAIASNIGGKQALETVQRLLPVLCQAHGLTPAQVVAIASHDGGK<br>QALETVQRLLPVLCQDHGLTPDQVVAIASNGGGKQALETVQRLLPVLCQDHGLTPEQVVAIANNNGGKQALETVQ<br>RLLPVLCQAHGLTPDQVVAIASHDGGKQALETVQRLLPVLCQAHGLTPAQVVAIASNGGGKQALETVQRLLPVLC<br>QDHGLTPDQVVAIANNNGGKQALETVQRLLPVLCQDHGLTPEQVVAIASHDGGKQALETVQRLLPVLCQAHGLTP<br>DQVVAIASNGGGKQALETVQRLLPVLCQAHGLTPAQVVAIASHDGGKQALETVQRLLPVLCQDHGLTPDQVVAIA<br>SHDGGKQALETVQRLLPVLCQDHGLTPEQVVAIASHDGGKQALETVQRLLPVLCQAHGLTPDQVVAIANNNGGKQ<br>ALETVQRLLPVLCQAHGLTPAQVVAIASHDGGKQALETVQRLLPVLCQDHGLTPEQVVAIASNGGGRPALESIVA<br>QLSRPDPALAALTNDHLVALACLGGRPALDAVKKGLPHAPALIKRTNRRIPERTSHRVAGSDPKKKRKV*<br>His-tag (gray):3xFLAG tag (violet):SV40 nls(green):TALE[A](black):SV40<br>nls(green)
TALE[B]	MHHHHHHDYKDHDGDYKDHDIDYKDDDDKMAPKKKRKVGIHRGVPMVDLRTLGYSQQQEKIKPKVRSTVAQHHE<br>ALVGHGFTHAHIVALSQHPAALGTVAVKYQDMIAALPEATHEAIVGVGKQWSGARALEALLTVAGELRGPPLQLD<br>TGQLLKIAKRGGVTAVEAVHAWRNALTGAPLNLTPDQVVAIASHDGGKQALETVQRLLPVLCQDHGLTPEQVVAI<br>ASNGGGKQALETVQRLLPVLCQAHGLTPDQVVAIASNGGGKQALETVQRLLPVLCQAHGLTPAQVVAIASHDGGK<br>QALETVQRLLPVLCQDHGLTPDQVVAIASHDGGKQALETVQRLLPVLCQDHGLTPEQVVAIANNNGGKQALETVQ<br>RLLPVLCQAHGLTPDQVVAIASNGGGKQALETVQRLLPVLCQAHGLTPAQVVAIASNGGGKQALETVQRLLPVLC<br>QDHGLTPDQVVAIASNGGGKQALETVQRLLPVLCQDHGLTPEQVVAIASHDGGKQALETVQRLLPVLCQAHGLTP<br>DQVVAIASHDGGKQALETVQRLLPVLCQAHGLTPAQVVAIASNIGGKQALETVQRLLPVLCQDHGLTPDQVVAIA<br>SHDGGKQALETVQRLLPVLCQDHGLTPEQVVAIASNIGGKQALETVQRLLPVLCQAHGLTPDQVVAIASNGGGKQ<br>ALETVQRLLPVLCQAHGLTPAQVVAIASHDGGKQALETVQRLLPVLCQDHGLTPEQVVAIASNGGGRPALESIVA<br>QLSRPDPALAALTNDHLVALACLGGRPALDAVKKGLPHAPALIKRTNRRIPERTSHRVAGSDPKKKRKV*<br>His-tag (gray):3xFLAG tag (violet):SV40 nls(green):TALE[B](black):SV40<br>nls(green)
TALE[F]	MHHHHHHDYKDHDGDYKDHDIDYKDDDDKMAPKKKRKVGIHRGVPMVDLRTLGYSQQQEKIKPKVRSTVAQHHE<br>ALVGHGFTHAHIVALSQHPAALGTVAVKYQDMIAALPEATHEAIVGVGKQWSGARALEALLTVAGELRGPPLQLD<br>TGQLLKIAKRGGVTAVEAVHAWRNALTGAPLNLTPDQVVAIASHDGGKQALETVQRLLPVLCQDHGLTPEQVVAI<br>ASNIGGKQALETVQRLLPVLCQAHGLTPDQVVAIASNIGGKQALETVQRLLPVLCQAHGLTPAQVVAIASNGGGK<br>QALETVQRLLPVLCQDHGLTPDQVVAIASNIGGKQALETVQRLLPVLCQDHGLTPEQVVAIASNGGGKQALETVQ<br>RLLPVLCQAHGLTPDQVVAIANNNGGKQALETVQRLLPVLCQAHGLTPAQVVAIASNIGGKQALETVQRLLPVLC<br>QDHGLTPDQVVAIASHDGGKQALETVQRLLPVLCQDHGLTPEQVVAIASHDGGKQALETVQRLLPVLCQAHGLTP<br>DQVVAIANNNGGKQALETVQRLLPVLCQAHGLTPAQVVAIASNGGGKQALETVQRLLPVLCQDHGLTPDQVVAIA<br>SNIGGKQALETVQRLLPVLCQDHGLTPEQVVAIASNGGGKQALETVQRLLPVLCQAHGLTPDQVVAIANNNGGKQ<br>ALETVQRLLPVLCQAHGLTPAQVVAIASNGGGKQALETVQRLLPVLCQDHGLTPEQVVAIANNNGGRPALESIVA<br>QLSRPDPALAALTNDHLVALACLGGRPALDAVKKGLPHAPALIKRTNRRIPERTSHRVAGDPKKKRKV*<br>His-tag (gray):3xFLAG tag (violet):SV40 nls(green):TALE[A](black):SV40<br>nls(green)
TetR:VP16	MHHHHHHSRLDKSKVINSALELLNEVGIEGLTTRKLAQKLGVEQPTLYWHVKNKRALLDALAIEMLDRHHTHFCP<br>LEGESWQDFLRNNAKSFRCALLSHRDGAKVHLGTRPTEKQYETLENQLAFLCQQGFSLENALYALSAVGHFTLGC<br>VLEDQEHQVAKEERETPTTDSMPPLLRQAIELFDHQGAEPAFLFGLELIICGLEKQLKCESGSDPKKKRKVAPPT<br>DVSLGDELHLDGEDVAMAHADALDDFDLDMLGDGDSPGPGFTPHDSAPYGALDMADFEFEQMFTDALGIDEYGG*<br>His-tag (gray):TetR(black):SV40 nls(green):VP16(blue)

Gal4:VP16	MHHHHHHKLLSSIEQACDICRLKKLKCSKEKPKCAKCLKNNWECRYSPKTKRSPLTRAHLTEVESRLERLEQLFL<br>LIFPREDLDMILKMDSLQDIKALLTGLFVQDNVNKDAVTDRLASVETDMPLTLRQHRISATSSSEESSNKGQRQL<br>TVSDPKKKRKVAPPTDVSLGDELHLDGEDVAMAHADALDDFDLDMLGDGDSPGPGFTPHDSAPYGALDMADFEFE<br>QMFTDALGIDEYGG*<br>His-tag (gray):Gal4(black):SV40 nls(green):VP16(blue)
Gal4	MHHHHHHKLLSSIEQACDICRLKKLKCSKEKPKCAKCLKNNWECRYSPKTKRSPLTRAHLTEVESRLERLEQLFL<br>LIFPREDLDMILKMDSLQDIKALLTGLFVQDNVNKDAVTDRLASVETDMPLTLRQHRISATSSSEESSNKGQRQL<br>TVSDPKKKRKV<br>His-tag (gray):Gal4(black):SV40 nls(green)
Zif268:VP16	MHHHHHHPGEKPYACPVESCDRRFSRSDELTRHIRIHTGQKPFQCRICMRNFSRSDHLTTHIRTHTGEKPFACDI<br>CGRKFARSDERKRHTKIHTGDPKKKRKVAPPTDVSLGDELHLDGEDVAMAHADALDDFDLDMLGDGDSPGPGFTP<br>HDSAPYGALDMADFEFEQMFTDALGIDEYGG*<br>His-tag (gray):Zif268(black):SV40 nls(green):VP16(blue)
Zif268	MHHHHHHPGEKPYACPVESCDRRFSRSDELTRHIRIHTGQKPFQCRICMRNFSRSDHLTTHIRTHTGEKPFACDI<br>CGRKFARSDERKRHTKIHTGDPKKKRKV<br>His-tag (gray):Zif268(black):SV40 nls(green)
TetR:KRAB	MHHHHHHSRLDKSKVINSALELLNEVGIEGLTTRKLAQKLGVEQPTLYWHVKNKRALLDALAIEMLDRHHTHFCP<br>LEGESWQDFLRNNAKSFRCALLSHRDGAKVHLGTRPTEKQYETLENQLAFLCQQGFSLENALYALSAVGHFTLGC<br>VLEDQEHQVAKEERETPTTDSMPPLLRQAIELFDHQGAEPAFLFGLELIICGLEKQLKCESGSDPKKKRKVKDVF<br>VDFTREEWKLLDTAQQIVYRNVMLENYKNLVSLGYQLTKPDVILRLEKGEEPWLVEREIHQETHPDSETAFEIKS<br>SV*<br>His-tag (gray):TetR(black):SV40 nls(green):KRAB(magenta)
Gal4:KRAB	MHHHHHHKLLSSIEQACDICRLKKLKCSKEKPKCAKCLKNNWECRYSPKTKRSPLTRAHLTEVESRLERLEQLFL<br>LIFPREDLDMILKMDSLQDIKALLTGLFVQDNVNKDAVTDRLASVETDMPLTLRQHRISATSSSEESSNKGQRQL<br>TVSDPKKKRKVKDVFVDFTREEWKLLDTAQQIVYRNVMLENYKNLVSLGYQLTKPDVILRLEKGEEPWLVEREIH<br>QETHPDSETAFEIKSSV*<br>His-tag (gray):Gal4(black):SV40 nls(green):KRAB(magenta)
Zif268:KRAB	MHHHHHHPGEKPYACPVESCDRRFSRSDELTRHIRIHTGQKPFQCRICMRNFSRSDHLTTHIRTHTGEKPFACDI<br>CGRKFARSDERKRHTKIHTGDPKKKRKVPKKKRKVDGGGALSPQHSAVTQGSIIKNKEGMDAKSLTAWSRTLVTF<br>KDVFVDFTREEWKLLDTAQQIVYRNVMLENYKNLVSLGYQLTKPDVILRLEKGEEPWLVEREIHQETHPDSETAF<br>EIKSSV*<br>His-tag (gray):Zif268(black):SV40 nls(green):KRAB(magenta)
dCas9	MHHHHHHDKKYSIGLAIGTNSVGWAVITDEYKVPSKKFKVLGNTDRHSIKKNLIGALLFDSGETAEATRLKRTAR<br>RRYTRRKNRICYLQEIFSNEMAKVDDSFFHRLEESFLVEEDKKHERHPIFGNIVDEVAYHEKYPTIYHLRKKLVD<br>STDKADLRLIYLALAHMIKFRGHFLIEGDLNPDNSDVDKLFIQLVQTYNQLFEENPINASGVDAKAILSARLSKS<br>RRLENLIAQLPGEKKNGLFGNLIALSLGLTPNFKSNFDLAEDAKLQLSKDTYDDDLDNLLAQIGDQYADLFLAAK<br>NLSDAILLSDILRVNTEITKAPLSASMIKRYDEHHQDLTLLKALVRQQLPEKYKEIFFDQSKNGYAGYIDGGASQ<br>EEFYKFIKPILEKMDGTEELLVKLNREDLLRKQRTFDNGSIPHQIHLGELHAILRRQEDFYPFLKDNREKIEKIL<br>TFRIPYYVGPLARGNSRFAWMTRKSEETITPWNFEEVVDKGASAQSFIERMTNFDKNLPNEKVLPKHSLLYEYFT<br>VYNELTKVKYVTEGMRKPAFLSGEQKKAIVDLLFKTNRKVTVKQLKEDYFKKIECFDSVEISGVEDRFNASLGTY<br>HDLLKIIKDKDFLDNEENEDILEDIVLTLTLFEDREMIEERLKTYAHLFDDKVMKQLKRRRYTGWGRLSRKLING<br>IRDKQSGKTILDFLKSDGFANRNFMQLIHDDSLTFKEDIQKAQVSGQGDSLHEHIANLAGSPAIKKGILQTVKVV<br>DELVKVMGRHKPENIVIEMARENQTTQKGQKNSRERMKRIEEGIKELGSQILKEHPVENTQLQNEKLYLYYLQNG<br>RDMYVDQELDINRLSDYDVDAIVPQSFLKDDSIDNKVLTRSDKNRGKSDNVPSEEVVKKMKNYWRQLLNAKLITQ<br>RKFDNLTKAERGGLSELDKAGFIKRQLVETRQITKHVAQILDSRMNTKYDENDKLIREVKVITLKSKLVSDFRKD<br>FQFYKVREINNYHHAHDAYLNAVVGTALIKKYPKLESEFVYGDYKVYDVRKMIAKSEQEIGKATAKYFFYSNIMN<br>FFKTEITLANGEIRKRPLIETNGETGEIVWDKGRDFATVRKVLSMPQVNIVKKTEVQTGGFSKESILPKRNSDKL<br>IARKKDWDPKKYGGFDSPTVAYSVLVVAKVEKGKSKKLKSVKELLGITIMERSSFEKNPIDFLEAKGYKEVKKDL<br>IIKLPKYSLFELENGRKRMLASAGELQKGNELALPSKYVNFLYLASHYEKLKGSPEDNEQKQLFVEQHKHYLDEI<br>IEQISEFSKRVILADANLDKVLSAYNKHRDKPIREQAENIIHLFTLTNLGAPAAFKYFDTTIDRKRYTSTKEVLD<br>ATLIHQSITGLYETRIDLSQLGGDDPKKKRKV*<br>His-tag (gray):dCas9(black):SV40 nls(green)

Table S5: RNA seqence used in this study

**LABEL**	**SEQUENCE**
gRNA[At]	tttactgctgctcccgctactgttttagagctagaaatagcaagttaaaataaggctagtccgttatcaacttga<br>aaaagtggcaccgagtcggtgcttttttt<br>Guiding sequence(black):gRNA scaffold(green)

Table S6: *In silico* analysis of TALE[A] and Zif:VP16 on indicated reporters

	**A:5nt:zif**	**A:20nt:zif**
Total models	150	150
Homology models (% of clashes)	62%	13%
Free models (% of clashes)	64%	4%

Table S7: Reporter sequences other than luc2CP used in this study. Sequence was introduced to pGL4.16 vector right after sequence A:tet (Table S2).

LABEL	SEQUENCE
BFP	MSELIKENMHMKLYMEGTVDNHHFKCTSEGEGKPYEGTQTMRIKVVEGGPLPFAFDILATSFLYGSKTFINHTQG<br>IPDFFKQSFPEGFTWERVTTYEDGGVLTATQDTSLQDGCLIYNVKIRGVNFTSNGPVMQKKTLGWEAFTETLYPA<br>DGGLEGRNDMALKLVGGSHLIANIKTTYRSKKPAKNLKMPGVYYVDYRLERIKEANNETYVEQHEVAVARYCDLP<br>SKLGHKLNGTNSACKNWFSSLSHFVIHLNSHGFPPEVEEQAAGTLPMSCAQESGMDRHPAACASARINV*<br>BFP (blue):CL1 PEST signal (gray)

Table S8: P-values corresponding to statistical analysis of samples with tet reporter and TetR:VP16 in Figure 4B with and without added TALE[A].

LABEL ON GRAPH	P-value
0 bp	0.0004
5 bp	1.6E-08
20 bp	2.7E-05
40 bp	0.0023
80 bp	0.0015
100 bp	3.6E-05
250 bp	0.2301

Table S9: P-values corresponding to statistical analysis of samples with gal reporter and Gal4:VP16 in Figure 5B with and without added TALE[A].

LABEL ON GRAPH	P-value
0 bp	0.0002
5 bp	0.0007
20 bp	3.6E-06
40 bp	0.0013
80 bp	0.0005
100 bp	0.0013
250 bp	0.2738

Table S10: P-values corresponding to statistical analysis of samples with zif reporter and Zif268:VP16 in Figure 5E with and without added TALE[A].

LABEL ON GRAPH	P-value
0 bp	6.94E-07
5 bp	0.0050
20 bp	0.0093
40 bp	0.0012
80 bp	2.4E-06
100 bp	0.0002
250 bp	0.2351
